# Diversity in the Interaction of Amino Acid- and Peptide-Based Hydroxamic Acids with Some Platinum Group Metals in Solution

**DOI:** 10.3390/molecules27030669

**Published:** 2022-01-20

**Authors:** Linda Bíró, Péter Buglyó, Etelka Farkas

**Affiliations:** Department of Inorganic and Analytical Chemistry, Faculty of Science and Technology, University of Debrecen, 4032 Debrecen, Hungary; linda.biro@science.unideb.hu (L.B.); buglyo@science.unideb.hu (P.B.)

**Keywords:** hydroxamic acids, platinum group metals, amino acid derivatives, metal complex solutions

## Abstract

Complexes that incorporate both ligand(s) and metal(s) exhibiting cytotoxic activity can especially be interesting to develop multifunctional drug molecules with desired activities. In this review, the limited number of solution results collected in our laboratory on the complexes of Pd(II) and two other platinum group metals—the half-sandwich type, [(η^6^-*p*-cym)Ru(H_2_O)_3_]^2+^, and [(η^5^-Cp*)Rh(H_2_O)_3_]^2+^—with hydroxamic acid derivatives of three amino acids, two imidazole analogues, and four small peptides are summarized and evaluated. Unlike the limited number of coordination sites of these metal ions (four and three for Pd(II) and the organometallic cations, respectively), the ligands discussed here offer a relatively high number of donor atoms as well as variation in their position within the ligands, resulting in a large versatility of the likely coordination modes. The review, besides presenting the solution equilibrium results, also discusses the main factors, such as (N,N) versus (O,O) chelate; size of chelate; amino-N versus imidazole-N; primary versus secondary hydroxamic function; differences between hydrolytic ability of the metal ions studied; and hydrolysis of the coordinated peptide hydroxamic acids in their Pd(II) complexes, which all determine the coordination modes present in the complexes formed in measurable concentrations in these systems. The options for the quantitative evaluation of metal binding effectivity and selectivity of the various ligands and the comparison with each other by using solution equilibrium data are also discussed.

## 1. Introduction

For many reasons, investigation of the metal-binding properties of hydroxamic acid-based organic bioligands (containing at least one hydroxamic acid function, R_C_C(O)R_N_N(OH), where R_C_ = alkyl or aryl; R_N_ = H in primary derivatives, alkyl or aryl in secondary ones), whether naturally occurring or synthetic, has been a focus of interest for many decades. Most of the results are summarized in excellent reviews, some of which are referred here [1,2,3,4,5,6,7,8,9,10,11,12,13,14,15,16,17,18,19,20]. The previous results clearly demonstrate the significance of hydroxamate-based compounds in many fields, for example, they are known to play a crucial role in microbial iron uptake [3,5,6,11] and are capable of inhibiting a variety of enzymes, including ureases, peroxidases, and matrix metalloproteinases [8,9,10,14,16,18,19,20,21,22,23,24,25]. In correlation with these biological roles/effects, hydroxamate-based compounds are frequently used in the design of therapeutics targeting different diseases, like iron overload, cancers, microbial infections, inflammations, etc. [8,9,10,26,27,28,29,30,31,32,33]. A further feature of them is the ability to release NO under certain conditions [12,14]. In most of the cases, the biological roles and effects of the hydroxamate-based compounds are in direct correlation with their strong chelating ability. Therefore, the study of their interaction with metal ions has a permanent interest in numerous laboratories. As is already well-known, hydroxamates typically coordinate to metal centers through the carbonyl oxygen atom and the deprotonated hydroxyl group, resulting in the formation of a 5-membered (O,O)-type hydroxamato-chelate. Under special conditions however, a few other modes are also known, e.g., binding through the deprotonated nitrogen [12,14,20]. Furthermore, the complexation behaviour of hydroxamic acids can significantly be altered by the presence of additional donors, such as amino group(s) in amino acid derivatives or amino and peptide-amide moieties in peptide derivatives [12,20,34].

**Scheme 1:**
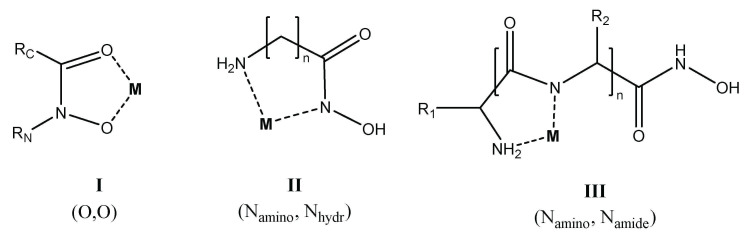
Some typical binding modes in simple hydroxamate, amino-, or peptide hydroxamate complexes.

As it is seen in Scheme 1, in addition to the hydroxamate-type (O,O) chelate (structure I), even if the parent amino acid does not have a side-chain donor, primary derivatives of α-, β- and γ-amino acids are capable of forming 5-, 6-, and 7-membered (N,N)-type chelates (structure II), respectively, via the amino-*N* and deprotonated hydroxamate or doubly deprotonated hydroximate-N atoms [13,34]. The versatility of the coordination modes can be even higher with amino acids containing side chain donors [34,35,36], or with peptide derivatives (structure III) [37,38,39,40,41].

However, the actually implemented coordination mode is also very much dependent on additional factors, e.g., the properties of the metal ion, pH, etc. All the previous results support that with hard metal ions only (O,O)-coordination occurs, while the soft ones prefer the nitrogen donors [34]. The most interesting binding modes, however, can occur with borderline metal ions, with which the formation of both types of chelates is likely, and this, in many cases, results in the formation of exciting polynuclear coordination complexes with high structural and functional diversity. In particular, metallacrowns (MCs, incorporating metal-heteroatom coordination units, (–[M–N–O]–)_n_), in a cyclic arrangement) have been attracting considerable attention due to their fascinating molecular architecture and diverse applicability, such as molecular magnets, catalysts, sensors, and recognition reagents for cations, anions, or molecules [40,41,42,43,44,45,46,47,48,49]. Although, among the ligands, amino acid-based hydroxamates are frequently used for producing metallacrowns, there are examples for the existence of MCs with other types of ligands, including peptide-based hydroxamic acids as well [40,41]. Most of the published homometallic metallacrowns incorporate 3*d* metal ions, while heterometallic ones commonly contain a 3*d* metal ion in the ring and a 4*f* ion as a core metal, but, rarely, some metallacrowns with other types of metal ions have also been synthesized [43,44]. For example, trinuclear metallamacrocycles containing half-sandwich complexes of Ru(II), Rh(III), and Ir(III) with bidentate pyridinonate ligands have also been reported for their potential application as chemosensors [50], while trinuclear macrocyclic structures consisting of half-sandwich type Ru(II) and some amino acidate ligands were developed and tested as catalysts [51]. However, interaction of aminohydroxamic acids with these half-sandwich type cations in solution have only been investigated in some of our recent works [52,53]. Furthermore, only a few works are published on Pt(II)–aminohydroxamates supporting Pt(II)-assisted hydrolysis of the coordinated ligands [54,55].

Given that a number of enzymes require metal ions for their catalytic activity, metal chelation-based metalloenzyme inhibition has become one of the strategies for the treatment of diseases. Therefore, metalloenzyme inhibitory effects of hydroxamate-based chelators have also induced accelerated interest in the past several decades [8,12,18,19,22,23,24,25,29,56]. Excellent reviews demonstrate that in many works, numerous hydroxamate-based compounds as potential therapeutical agents, especially for cancer therapy, have been developed [8,9,18,19,33,57]. Furthermore, numerous works have been induced by the fact/idea that the enhancement of selectivity and effectivity of the developed organic biomolecules can be achieved via incorporating them into metal complexes [33,51,58]. Despite the widespread use of platinum-based chemotherapeutic agents, as well as the great interest to develop new drug candidates with platinum [59,60,61,62] and other platinum group metals [61,62,63,64,65,66,67,68,69,70,71,72,73], it is surprising that there have been very few solution equilibrium results published for complexes with peptidehydroxamate- and aminohydroxamate-based chelators [74,75]. This low interest is most likely due to the inertness of complexes formed with platinum group metals, as well as the high tendency of these metal ions for hydrolysis [76]. However, as it is well-known, the rate of complex formation, namely, the “lability” or “inertness” of a complex, is very much determined by the mobility of the solvent molecules coordinated to the metal ion and can also be tuned by the nature of the ligands replacing the coordinated water molecule(s) [77]. Taking the advantage of these opportunities, several relevant solution equilibrium works have been performed in our laboratory. The present review summarizes our results on the interaction of the hydroxamic acid derivatives of amino acids and peptides with platinum group metals and focuses on the factors, which can affect the metal-binding properties of such organic ligands and, at the same time, their likely biological activity and metal ion selectivity. Some comparison is also presented between the metal binding effectivity of the ligands discussed here and some small (N,N), (N,O), (O,O), and (S,O) chelators.

## 2. Competitive Processes of the Metal-Ligand Interactions

### 2.1. Protonation Processes of the Ligands

Although hydroxamic acid-based compounds have been known for a very long time and are widely used in numerous fields [5,8,9,10,11,12,13,14,15,16,17,18,19,20,21,22,23,24,25,26,27,28,29,30,31,32,33] due to the ability of this function to adopt several forms [13,34], even recently, some new information for the acid–base properties of a hydroxamic function has come to light [78,79]. There is no doubt that this function (even a primary one) releases one dissociable proton, and this process leads to the formation of the monoanionic hydroxamate function. Usually, the pH-range of the deprotonation is ca. 7–10, but, for a certain molecule, it depends on various factors. For example, there is always a significant increase in the acidities (decrease in the p*K*) of secondary derivatives compared to when the corresponding primary counterparts are detected [80]. Under certain conditions with primary derivatives, further metal ion-induced deprotonation of the monoanionic function (formation of a hydroximate dianion) is also known [21].

**Scheme 2:**
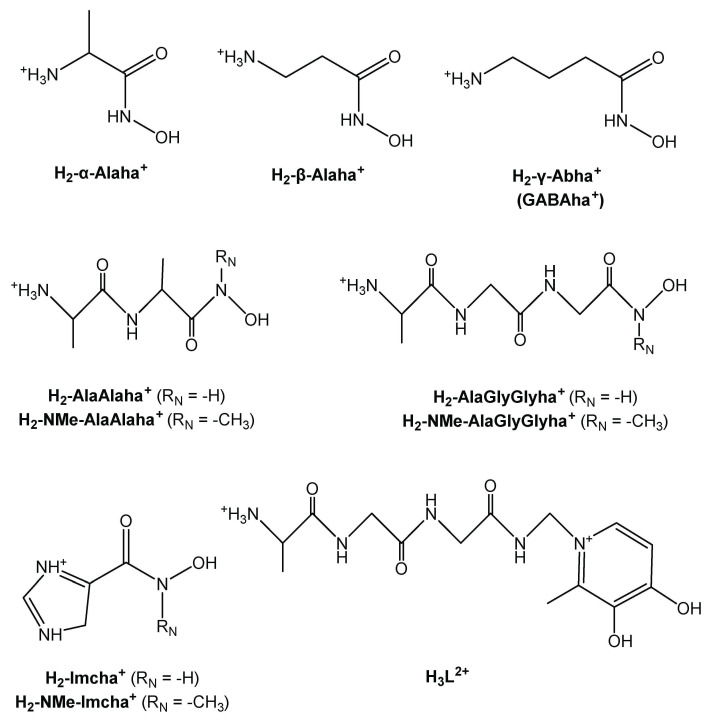
Structures of the fully protonated form of the ligands discussed.

Beside the hydroxamic group, each of the fully protonated ligands discussed here incorporates another weak acidic function and ammonium moiety in aminohydroxamic and peptidehydroxamic acids—and imidazolium in the imidazole derivatives (Scheme 2). Consequently, these ligands are capable of releasing two protons from their fully protonated forms in the measurable pH-range.

**Figure 1:**
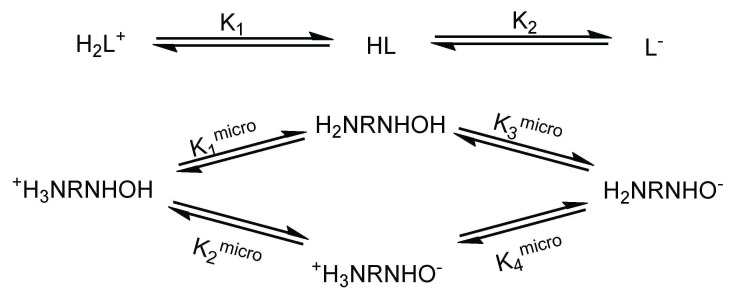
Macro and micro proton dissociation processes of aminohydroxamates together with the appropriate constants; for the values see Table 1.

Predominantly pH-potentiometric titrations, which are often ^1^H NMR, while in a few cases they are ^13^C NMR, have been used to determine the corresponding dissociation constants [34,37,81,82]. The pH-potentiometric results provide macro constants (p*K*_1_ and p*K*_2_ in Table 1 and Table 3), which cannot be assigned unambiguously to either of the acidic groups; they characterize the ligands at the macro level (Figure 1). However, the macroscopic dissociation constants in Table 1 clearly show the pH-range, where the (de)protonation processes compete with the metal–ligand complexation processes (up to pH ca. p*K* + 1).

**Figure 2:**
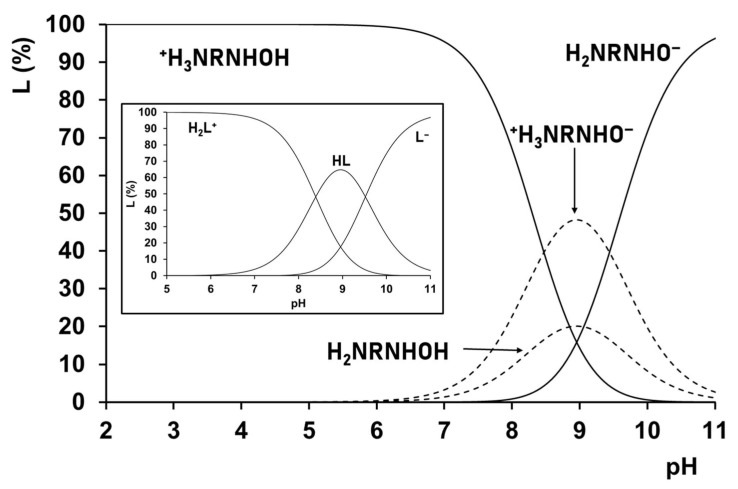
Concentration distribution curves of the proton complexes of H_2_-β-Alaha^+^, calculated using the dissociation macro constants (inlet) and micro constants as a function of pH. Dash lines refer to the corresponding binding isomers of HL.

For the imidazole derivatives, NMR results clearly supported that the lower dissociation constants belong to the deprotonation of the imidazolium-N(3)H^+^, while the higher ones belong to the hydroxamic functions. Moreover, the existence of hydrogen bonding between the non-protonated imidazole-N and hydroxamic-NH was supposed from the surprisingly low p*K*_1_ of H_2_-Imcha^+^ [81]. Unlike the imidazole derivatives, all of the results for the derivatives of the amino acids and peptides supported the significant overlapping of the two deprotonation steps [34,37,82,83]. In such cases, only the dissociation micro constants (p*K*^micro^), which can be determined by the selective monitoring of the dissociation of the individual group(s), and are characteristic for the individual processes shown in the heading of Table 1. As a representative example, the schematic presentation of the difference between the macroscopic and microscopic dissociation processes and pH-dependent speciation profile for H_2_-β-Alaha^+^ are seen in Figure 2.

The micro constants have not been determined for all of the ligands discussed here; it was done for H_2_-α-Alaha^+^, H_2_-β-Alaha^+^ [82], and for H_2_-AlaAlaha^+^ [37] as well as, under slightly different conditions, for H_2_-γ-Abha^+^ [83], but the results allowed us to draw conclusions for the acidity trend of the two sites for all of the discussed molecules. For H_2_-α-Alaha^+^ and the peptidehydroxamic acid derivatives, the NH_3_^+^-moiety is the more acidic, while for H_2_-β-Alaha^+^ and H_2_-γ-Alaha^+^, the order is the opposite (see *K*_1_^micro^/*K*_2_^micro^ in Table 1).

### 2.2. Hydrolytic Behaviour of the Metal Ions Discussed

**Scheme 3:**
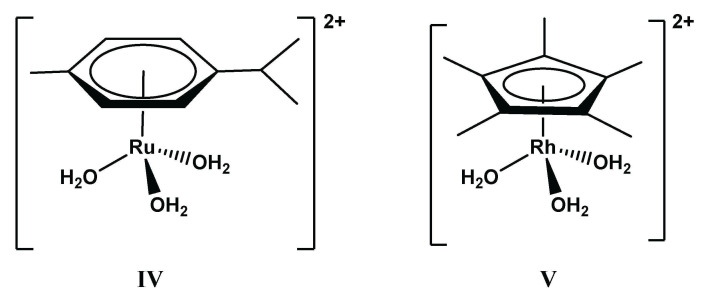
Structure of the half-sandwich type organometallic cations discussed.

Hydrolysis of the metal ions is also an important factor that needs to be taken into consideration as a competitive process during complex formation. Platinum group metals consist of the following 4*d* metals: ruthenium, rhodium, and palladium, as well as their heavier 5*d* congeners: osmium, iridium, and platinum, respectively. These metals in the form of +2 or +3 oxidation state ions are characterized by low stability aqua complexes that tend to hydrolyze heavily. Since the [M(H_2_O)_6_]^n+^ (M = Ru, Os, Rh, Ir; *n* = 2–3) or [M(H_2_O)_4_]^2+^ (M = Pd, Pt) cations are known to have slow or very slow and unexplored hydrolytic processes, no solution equilibrium studies can be found in the literature on their complexation processes in aqueous solution. Notably, among the monodentate ligands, halogenide ions—typically, chloride ions—can stabilize the metal ions in an aqueous medium in the form of octahedral or square planar chlorido complexes with moderate stability. Being a single exception, Pd(II) in the form of [PdCl_4_]^2−^, as a metal-containing component, can be involved in equilibrium studies. For the other metals with different forms or oxidation states, reliable solution equilibrium studies require the knowledge of their properly explored and quantified hydrolytic properties. Although both half-sandwich types, [(η^6^-*p*-cym)Ru(H_2_O)_3_]^2+^ and [(η^5^-Cp*)Rh(H_2_O)_3_]^2+^ (see Scheme 3), and the square-planar Pd(II) cations used in the studied systems and reviewed in this paper have a high tendency for hydrolysis, the presence of the strongly coordinating chloride ions can significantly influence the speciation, thereby hindering the formation of different hydroxido complexes.

In a chloride free medium, dissociation of one or two water molecules of the organometallic cations has been described, leading to the formation of [M_2_(OH)_2_]^2+^ and [M_2_(OH)_3_]^+^ hydroxido species [84,85], and the thermodynamic stability constants, of which are collected in Table 2. In the absence of chloride ions, the [(η^6^-*p*-cym)Ru(H_2_O)_3_]^2+^ starts to hydrolyze around pH 3, that is about 1.5 units more acidic than what was found for [(η^5^-Cp*)Rh(H_2_O)_3_]^2+^. The tendency is the same in the presence of chloride ions, [(η^6^-*p*-cym)Ru(H_2_O)_3_]^2+^ is less resistant to hydrolysis due to its higher affinity towards O-donors. However, chloride ions as competitors are able to shift the hydrolytic processes occurring at higher pH. In the presence of chloride ions, formation of chlorido, hydroxido, and mixed chlorido–hydroxido species have been described for the Ru(II)-containing system, and their thermodynamic stability constants (log*β*) have been determined by pH-potentiometry and ^1^H NMR [84]. The effect of the chloride ions can also be taken into account by using the conditional stability constants (logβ*) in Table 2 that are strictly valid in a 0.20 M KCl medium.

**Figure 3:**
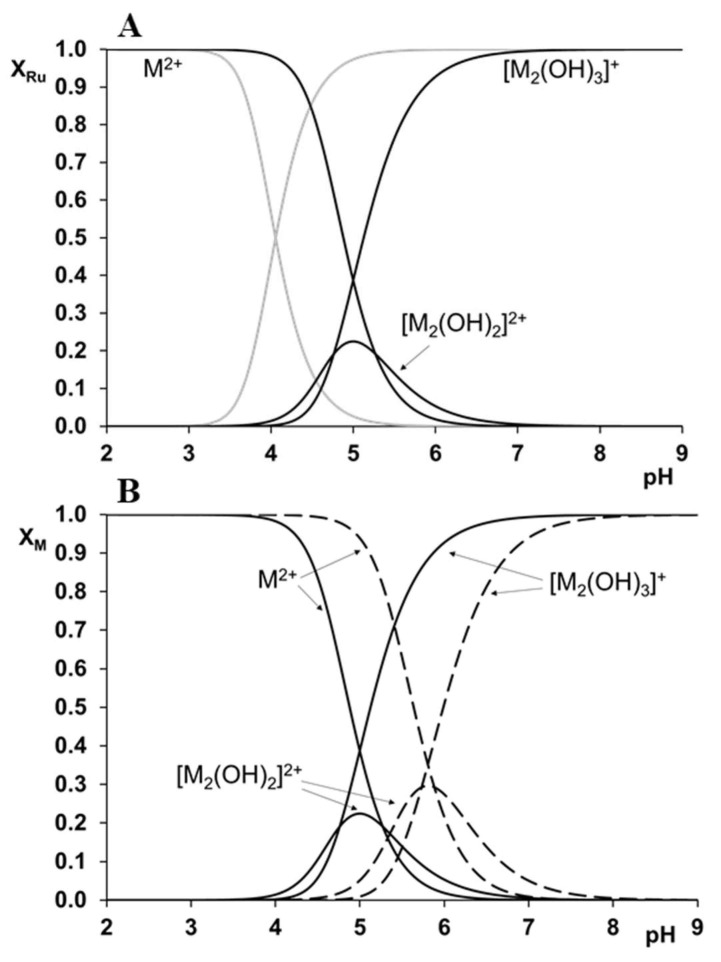
Concentration distribution curves calculated for (**A**) [(η^6^-*p*-cym)Ru(H_2_O)_3_]^2+^ at I = 0.20 M KNO_3_ (grey) and at 0.20 M KCl (black) and (**B**) [(η^6^-*p*-cym)Ru(H_2_O)_3_]^2+^ at I = 0.20 M KCl (continuous lines) and [(η^5^-Cp*)Rh(H_2_O)_3_]^2+^ at I = 0.20 M KNO_3_ (dashed lines). (M stands for [(η^6^-*p*-cym)Ru]^2+^ or [(η^5^-Cp*)Rh]^2+^, t = 25.0 °C, c_M_ = 1 mM)].

Although changing the supporting electrolyte from KNO_3_ to KCl—the latter one has more biological relevance—can result in a shift of the hydrolysis of both the organometallic cations about 1 pH unit to the higher pH values, at pH = 7.4 the dominance of the hydrolytic species is still observed. This is demonstrated in Figure 3A by the concentration distribution curves calculated for [(η^6^-*p*-cym)Ru(H_2_O)_3_]^2+^ using the two abovementioned supporting electrolytes, and by a comparison between Figure 3A,B. Figure 3B clearly indicates the more preferred hydrolysis of [(η^6^-*p*-cym)Ru(H_2_O)_3_]^2+^ even in the presence of chloride ions when compared to that of [(η^5^-Cp*)Rh(H_2_O)_3_]^2+^ in the presence of nitrate.

The study of the hydrolytic properties of the corresponding 5*d* analogues, the [(η^6^-*p*-cym)Os(H_2_O)_3_]^2+^ and [(η^5^-Cp*)Ir(H_2_O)_3_]^2+^ aqua cations, has also been carried out at different ionic strengths [86], but, due to their slow complex formation with (N,N)-donors, no appreciable equilibrium results have been found with either aminohydroxamic or peptidehydroxamic acids. Therefore, the hydrolytic behaviour of these 5*d* cations is not discussed here.

Platinum(II) complexes are widely used in chemotherapy [87], however, solution studies are hindered due to the kinetic inertness of most of the complexes of this metal ion. To overcome this problem, and to get a deeper insight into the interactions that may occur with various bioligands, the more labile palladium(II) is used as a model. The simple palladium(II) aqua ion, [Pd(H_2_O)_4_]^2+^, is not stable and is extensively hydrolyzed in acidic solution; the equilibrium quotients of the [Pd(OH)]^+^ and [Pd(OH)_2_] complexes have been determined in earlier works [88]. Hydrolysis can be suppressed by using the monofunctional or bifunctional derivatives of [Pd(H_2_O)_4_]^2+^ as metal ion sources, in which two or three coordination sites of the square-planar palladium are occupied by strongly coordinating N-donors, such as diethylenetriamine (dien), terpyridine (terpy), ethylenediamine (en), or 2-picolylamine (pic).

It is well-known from the literature that the free palladium(II) forms stable complexes with numerous ligands—especially with N-donors—below pH 2, resulting in difficulties in the determination of the stability constants [89]. However, complex formation can be shifted in the measurable pH-range using [PdCl_4_]^2−^ as a metal ion source. Due to the high excess of chloride ions as competitive ligands, hydrolysis of the metal ion becomes completely hindered under biologically relevant conditions. While the hydrolysis can be excluded at 0.20 M KCl, the effect of Cl^−^ is not negligible. log*β* values for the complexes formed in the Pd(II)–Cl^−^ system are reported in the literature and are as follows: [PdCl]^+^ = 4.47, [PdCl_2_] = 7.76, [PdCl_3_]^−^ = 10.17, and [PdCl_4_]^2−^ = 11.54 [90].

## 3. Interaction between Platinum Group Metal Ions and Amino-, Peptidehydroxamates or Imidazole-Containing Analogous Ligands

The low oxidation state of Ru-Os and Rh-Ir pairs can be stabilized in organometallic half-sandwich type cations too, which are very often constituents of complexes with proven anticancer potential. In general, the [(η^6^-arene)M(H_2_O)_3_]^2+^ (M = Ru, Os) or [(η^5^-arenyl)M(H_2_O)_3_]^2+^ (M = Rh, Ir) cations have high oxidative stability and are stable in water. Although the rather inert character of the osmium and iridium half-sandwich type complexes hinders the study of their complexation processes in most cases, for the ruthenium- and rhodium-containing cations even conventional pH-potentiometry can be used. Since their hydrolytic processes have been explored and quantified in detail (as it was discussed in Section 2), speciation studies were also possible with amino acids, peptidehydroxamic acids, and imidazole-containing analogous ligands, as it will be shown in the next sections. For Pd(II), due to the previously discussed intensive hydrolysis of [PdCl_4_]^2−^, [Pd(en)]^2+^, [Pd(bpy)]^2+^, or similar compounds, which can be used as a metal precursor, the latter cations of the Pd-N bonds are expected to be very inert, and therefore remain unaltered during complex formation with other ligands.

### 3.1. Evaluation of the Stability Data for the Ru/Rh Complexes with Aminohydroxamates, Peptidehydroxamates, or Imidazole-Containing Analogous Ligands

#### 3.1.1. Factors Determining the Bonding Modes, Equilibrium Models, and Stability of the Ru/Rh Complexes with Aminohydroxamates, Peptidehydroxamates, or Imidazole-Containing Analogous Ligands

Conventional pH-potentiometric analysis of the titration curves registered at various metal ion-to-ligand ratios yielded the models and stability constants published in references [52,53,75,91] which are summarized in Table 3.

In solution equilibrium studies on bio-relevant systems, a KCl (or NaCl) electrolyte is frequently used for maintaining the ionic strength constant. The discernable interaction between these organometallic cations and the chloride ion (see Section 2) suggests the fact that Cl^−^ acts as a competitor of the ligands in these systems and has motivated the use of another supporting electrolyte, e.g., KNO_3_, in many cases. However, as it is seen in Table 3 for H_2_-γ-Abha^+^, the stability constants were determined at 0.20 M KNO_3_ and the KCl (bold) ionic strength was determined too. Comparison of these data sets reveals that, in the presence of chloride ions, the obtained log*β* values are always smaller than the corresponding ones calculated for systems where the ionic strength was set up with KNO_3_. This difference clearly indicates a measurable interaction of these organometallic cations with the chloride ion. Consequently, a simple comparison of the equilibrium data in Table 3 in the presence of the two different electrolytes does not allow for the drawing of any conclusion on the effectivity differences of the metal–ligand interactions.

The missing data in Table 3 for some systems indicate that pH-potentiometry turned out to be unsuitable for registering reliable titration curves and carrying out subsequent calculations due to the slow complex formation processes. Furthermore, in the Ru-Imcha^−^ system, the formation of precipitate over a wide pH-range also hindered the determination of the speciation model.

The models in Table 3 are the results of various calculations and the data sets yielding the smallest fitting parameter and are supported by independent NMR and ESI-TOF-MS results, which were accepted as the final ones.

The models in Table 3 share similarities with each other and consist of mono- and dinuclear species, the latter being major ones in many cases. The reason behind this is that, for all the ligands discussed here, the number of donor atoms available for coordination is greater than three, while the two studied organometallic cations have only three coordination sites accepting donor atoms from the ligands. Moreover, due to steric reasons, none of these ligands are capable of coordinating to all three of the sites of a single metal cation. Instead, two sites are occupied by a certain coordinating ligand via a chelate (either (O,O), (N,N), or (N,O), depending on the relative stability of them) and the third one is occupied by a solvent molecule, hydroxide, chloride ion (if present), or one donor from an additional ligand, which often results in the formation of di/oligonuclear species with all these hydroxamic-based compounds. Also, the hydrogen ion binding ability of these types of ligands might affect their self-assembly (vide infra). Consequently, the identical stoichiometry for a given species with different ligands may indicate that they have an identical bonding mode but may possess a different coordination pattern too.

**Figure 4:**
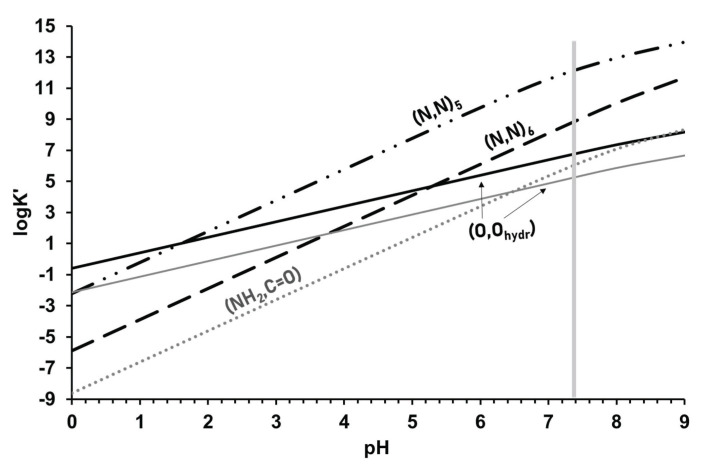
pH-dependence of the log*K*’ conditional stability constants at 0.20 M KNO_3_ for a model 5-membered and 6-membered (N,N) chelate (formed with en and pn, respectively), as well as for a 5-membered (O,O) (formed with aha^−^ at 0.20 M KNO_3_ or at 0.20 M KCl) and for a 5-membered (N_amino_,O_carb_) chelate (formed with GlyGlyAla^−^ at 0.20 M KCl) in their interaction with [(η^5^-Cp*)Rh]^2+^. Black lines indicate logK’ values determined in KNO_3_, while grey lines, values obtained in KCl medium. Vertical line indicates pH = 7.4.

To explore the most plausible binding modes, various experimental techniques (mostly NMR, UV-VIS, and ESI-TOF-MS) as well as analysis of the log*β* values were used. The latter can rely on the calculation and comparison of the conditional stability constants. Conditional constants, in which the competition between the proton and metal ions for the ligand is taken into account, can be calculated for a metal ion–model ligand system where the complex has a given (O,O) or (N,N) chelate that can model a chelating site for a ligand capable of coordinating via different donor sets. As an example, Figure 4 shows the calculated conditional stability constants for a model 5-membered (N,N) (en) and a 6-membered (N,N) (pn), as well as a 5-membered (O,O) (Haha) and a 5-membered (N_amino_,O_carb._) (HGlyGlyAla)-chelated system with [(η^5^-Cp*)Rh]^2+^ in the function of pH to predict the pH-dependent metal ion binding strengths of the available chelating sets of the aminohydroxamates and peptidehydroxamates. (The corresponding stability constants and protonation constants were taken from refs. [53], [92] and [93], except for the (O,O) chelated species at 0.20 M KCl, which were determined in this work and are as follows: [ML]^+^ = 7.08; [MH_−1_L]= −1.12). As Figure 4 reveals, in the strongly acidic pH-range (1–3) the stability of the (N,N)_5_ chelate is comparable with that of the (O,O) chelate, but upon increasing the pH, the latter becomes the most stable in the whole pH-range. The 6-membered (N,N)_6_ chelate has a significantly smaller stability compared to that of the (N,N)_5_ chelate across the whole pH-range. Its stability in the highly acidic pH-range is measurably smaller than that of the (O,O) chelate (obtained in the presence of KNO_3_) and is comparable to it within the range pH ca. 4–6, while it becomes more stable above pH 7.

Among the aminohydroxamic acid derivatives, in agreement with the theoretically predicted trends for H_2_-α-Alaha^+^, the complexation starts with the formation of [M_2_H_−1_L]^2+^ having mixed with the (O,O) and (N_amino_,N_hydr_) chelates (the ligand links two metal ions via the binding modes shown in Scheme 1, structures I and II). Above pH 3 the dinuclear complex is only present when there is an excess of metal ions. At a 1:1 ratio, the (N_amino_,N_hydr_) chelate remains unaltered, yielding [ML]^+^ (structure II) while the hydroxamate moiety is crowded out from coordination in line with the relative stability of the appropriate chelates. A further increase in pH enhances the deprotonation of the water molecule at the third coordination site, resulting in the formation of [MH_−1_L].

For the beta derivative, in line with the predictions too, the interaction starts with an (O,O)-chelated [MHL]^2+^ species but, upon increasing the pH, the 6-membered (N_amino_,N_hydr_) binding mode of the ligand can also be detected. As a result, [M_2_H_−1_L]^2+^ in this system is a major complex over a wide pH range, even at a 1:1 metal ion-to-ligand ratio, since the relative stability of the abovementioned two chelates are comparable (see Figure 4).

In the case of the γ-Abha system, experimental data support the formation of the hydroxamate (O,O)-chelated species under acidic conditions and, since the 7-membered (N_amino_,N_hydr_) chelate has significantly lower stability than that of the (O,O) chelate, its concurring effect is also smaller, resulting in the formation of much less of an amount of [M_2_H_−1_L]^2+^ than for the beta-derivative (as it is detailed in ref. [47]). It is also worth mentioning that [M_2_H_−1_L]^2+^ is the only species that has an identical binding mode with all three of the aminohydroxamates. Based on the data in Table 3 for this type of complex, the decreasing stability of the (N_amino_,N_hydr_) chelate with an increasing size is clearly reflected in the determined stability constants.

**Scheme 4:**
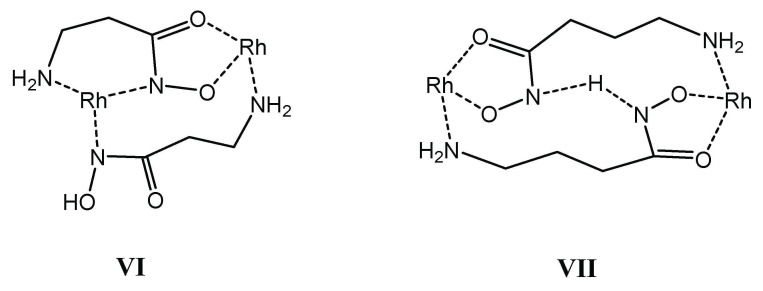
Suggested binding modes in [M_2_H_−1_L_2_]^+^ with the beta (VI) and gamma derivatives (VII); “Rh” stands for [(η^5^-Cp*)Rh]^2+^.

A common feature of the organorhodium–β-Alaha^–^ and –γ-Abha^−^ equilibrium models is the formation of 2:2 species with different protonation degrees too. However, despite the same composition ([M_2_L_2_]^2+^ and [M_2_H_−1_L_2_]^+^ are formed in both systems), the NMR and ESI-MS results supported significant differences between the binding modes of the complexes with the two ligands. To demonstrate this (as well as the strong H-bonding ability of a hydroxamic function), the binding modes for the [M_2_H_−1_L_2_]^+^ with the beta and gamma derivatives are shown in Scheme 4 in structures VI and VII, respectively.

In order to explore the effect of the type and basicity of the N-donor being in a chelatable position to the hydroxamate unit, the NH_2_ function in H_2_-α-Alaha^+^ was replaced by an imidazole ring with the synthesis of H-Imcha. Involvement of the secondary derivative, H_2_-NMe-Imcha^+^, into the studies provided further valuable information about the role of the hydroxamate-N in metal ion binding (formulas are shown in Scheme 2).

**Scheme 5:**
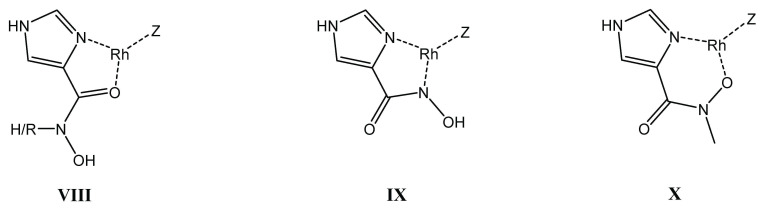
Some suggested binding modes for the imidazole hydroxamate complexes; “Rh” stands for [(η^5^-Cp*)Rh]^2+^; Z = Cl^−^/H_2_O, R = CH_3_ or H.

**Scheme 6:**
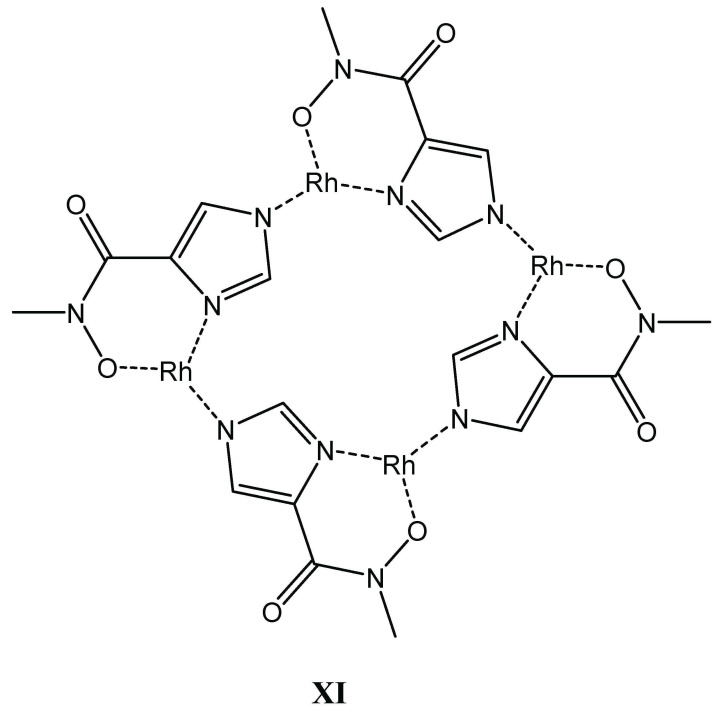
Suggested structure of the cyclic tetramer (XI) formed with the secondary imidazole hydroxamate; “Rh” stands for [(η^5^-Cp*)Rh]^2+^.

Although, for the organoruthenium cation, precipitation hindered the solution studies with the primary ligand and slow equilibria were detected with the secondary derivative, the organorhodium systems could be studied in detail. Similar to aminohydroxamates, the best models (Table 3) consist of various 1:1 and 2:1 species. However, because the proton is a significantly weaker competitor of the metal ion in the case of an imidazole-N compared to an amino-N (c.f. the corresponding p*K* values in Table 1 and Table 3), (O,O)-chelated species do not appear to a measurable extent with the imidazole derivatives. N_im_ is unambiguously the predominant donor (anchor) during the interactions, even under highly acidic conditions. With both ligands the complex formation starts with the involvement of the imidazole-N and the carbonyl-O donors (Scheme 5, structure VIII) in metal binding at low pH. Upon increasing the pH, a stable, 5-membered (N_im_,N_hydr_) chelate is present in the [ML] complex with the primary ligand (structure IX), while the binding mode is (N_im_,O_hydr_) with the secondary hydroxamate derivative forming a 6-membered chelate with lower stability (structure X). A further difference is that H-Imcha is capable of binding a second metal ion, linking them via its vacant hydroxamate function. In both systems NMR and ESI-MS evidences as well as for the secondary ligand DFT calculations supported the favoured formation of self-assembled oligonuclear complexes with [MH–1L]x general stoichiometry that have most likely cyclic structure. Moreover, the secondary ligand, imidazolato, units bridge the (N_im_,O_hydr_)-chelated metal cores due to the unavailability of the hydroxamate-N for coordination (as an example, see structure XI, Scheme 6) with the primary ligand, which is made of the highly stable (N_im_,N_hydr_)-chelated metal units that are most likely bridged by hydroxamate-O donors.

In peptidehydroxamates, the terminal amino group is already too far from the hydroxamate N or O atoms to form stable chelates. At the same time, these ligands contain at least one peptide bond that can also be involved in complex formation via the O_carb_ donor without deprotonation, or via the N_amide_ upon metal ion-assisted deprotonation and subsequent coordination. The results (discussed in ref. [75]) from the complexation of H_2_-AlaAlaha^+^, H_2_-NMeAlaAlaha^+^, H_2_-AlaGlyGlyha^+^, and H_2_-NMe-AlaGlyGlyha^+^ with the two organometallic half-sandwich type cations revealed that, in all cases, the interaction starts with the formation of a hydroxamate (O,O)-chelated species. This is in line with the conclusion that can be drawn from Figure 3. Namely, in the acidic pH range, the 5-membered (N_amino_,O_carb_) chelate has a much lower conditional stability than that of the (O,O) chelate and they become comparable with each other only above pH ca. 5, while the latter is slightly more stable above pH 6.5 (Given that the values of GlyGlyAla are valid at 0.20 M KCl, they can only be compared to the values of an (O,O) chelate calculated at 0.20 M KCl. Comparison of the pH-dependent logK’ values for the hydroxamate (O,O)-chelated species obtained in a KCl or KNO3 medium also highlights the concurring effect of the coordinating chloride ion with complex formation, resulting in lower logK’ values than in the presence of KNO3). Out of the dipeptide derivatives the primary H_2_-AlaAlaha^+^, being the (O,O) binding mode, remains in the whole studied pH-range as the (O,O) chelate is further strengthened by the deprotonation of the hydroxamate-N, yielding a hydroximato chelate. Consequently, although this ligand also incorporates strong donor nitrogens, those can only coordinate to a second metal ion. This results in dinuclear complexes (upon increasing the pH first with (N_amino_,O_carb_), then (N_amino_,N_hydr._) while above pH 6 stable (N_amino_,N_amide_,N_hydr._) binding modes) even at a 1:1 metal-to-ligand ratio.

**Scheme 7:**
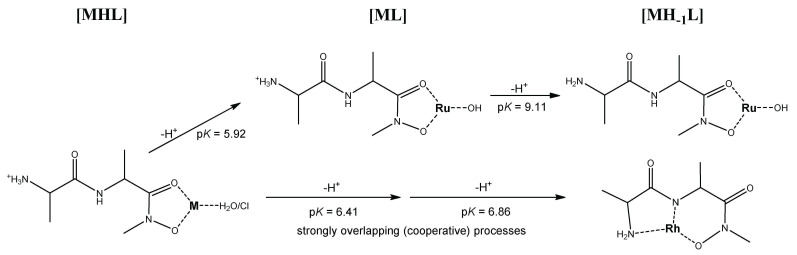
Comparison of the binding modes in the various Ru and Rh-NMe-AlaAla^−^ complexes ([MHL], [ML] and [MH_−1_L]); charges are omitted for clarity; “M” stands for one of the half-sandwich type metal ions, “Ru” for [η^6^-*p*-cym)Ru(H_2_O)_3_]^2+^, and “Rh” for [(η^5^-Cp*)Rh]^2+^.

Unavailability of the hydroxamate-N for coordination in the secondary H_2_-NMe-AlaAlaha^+^ results in the predominance of mononuclear complexes as well as practically identical equilibrium models with the two organometallic cations (Table 3). However, a comparison between these two systems in Scheme 7 demonstrates that the binding modes in the complexes formed with these two cations are rather different, due to the different tendencies of the two cations for hydrolysis (see Section 2.2).

As Scheme 7 shows, with [(η^6^-*p*-cym)Ru]^2+^, the hydrolysis of the coordinated water above pH ca. 5 occurs, which completely prevents the metal-induced deprotonation of the peptide-amide, and thus, also prevents the formation of the complex with an (N_amino_,N_amide_,O_hydr._) binding mode, which, in turn, is the dominating chelating set in the system with [(η^5^-Cp*)Rh]^2+^ above pH 7.

#### 3.1.2. Comparison of the Metal Binding Effectivity of the Ligands

In any condition where concurring equilibrium process(es) can occur simultaneously beside metal–ligand complexation, such as protonation process(es) of the ligand or hydrolysis of the metal ion, or both, the overall formation constants (log*β* values) do not correctly measure the effectiveness of a ligand to bind a certain metal ion [94]. Given that the coordinating donors of the ligands discussed here are protonated up to high pH (see Table 1), the metal ions must compete with protons for the binding sites over a wide pH-range (below the pH~p*K* + 1). Moreover, due to the high tendency of [η^6^-*p*-cym)Ru(H_2_O)_3_]^2+^ and [(η^5^-Cp*)Rh(H_2_O)_3_]^2+^ for hydrolysis (see Table 2), the ligands must compete with hydroxide ions for the metal ion above pH ca. 3 and pH ca. 5, respectively. To handle this situation, the effects of the concurring protonation and hydrolytic processes need to be taken into account. If the speciation profile is known, various calculations can be made to evaluate the effectiveness of a metal–ligand interaction: (i) The effect of the competitive processes can be considered by the conditional stability constant introduced by Schwarzenbach [95], which is valid for a given pH and under certain conditions, as it is shown for the model chelates in Figure 3. (ii) In bio-inspired studies, however, pM is generally used to measure the effectiveness of a ligand to bind a certain metal ion. pM = −log[M] (negative logarithm of the free metal ion concentration) as defined by Harris et al. and calculated for total c_L_ = 10^−5^ M and c_M_ = 10^−6^ M at pH 7.4 [96]. A higher numerical value of pM (lower concentration of the non-chelated metal ion) indicates a higher effectivity of the ligand to bind the metal ion. pM values were calculated for all of the discussed systems and are shown in Table 3. Although these values in Table 3 support the highest metal-binding effectivity of the imidazole derivatives and show clearly the α > β > γ effectivity order with amino acid derivatives, they indicate the stoichiometric binding of the two half-sandwich metal ions by almost all of the discussed ligands. The only exception is the [(η^5^-Cp*)Rh]^2+^–H_2_-γ-Abha^+^ system, where the pM = 6.4 value reveals the existence of a ca. 40% non-complexed metal ion under the mentioned conditions.

For these discussed pM values, the effect of the competition between the hydrogen ion and the metal ion for a certain ligand is handled correctly, but competition between the hydroxide ions and the ligand for the metal ion is not [13]. Due to the high tendency for hydrolysis of the metal ions discussed (see Section 2), the effect of metal ion hydrolysis on the metal–ligand interaction is expected. If there is a measurable competition between the ligand and hydroxide ion for the metal ion at physiological pH, the conditional pM’ can be an adequate parameter to evaluate the effectiveness of a ligand to bind the given metal ion. By definition, pM’ = −log[M]’ where [M’] = [M] + [M]_hydroxido complex_, (the total concentration of the metal ion non-complexed by the ligand). The pM’ values were also calculated and are presented in Table 3. A comparison between corresponding pM and pM’ values in Table 3 shows that (i) in the [(η^5^-Cp*)Rh]^2+^-containing systems pM~pM’, indicating that in these cases the hydroxide ion cannot compete with the ligands in a measurable extent. (ii) For the [(η^6^-*p*-cym)Ru]^2+^-containing systems, the pM~pM’ situation (lack of competition with the hydroxide ions) can only be seen with H_2_-NMe-Imcha^+^. In all of the other cases, pM ≠ pM’ with the latter being smaller, and, in some cases, significantly smaller than the former one. This means that in these systems, only the pM’ can correctly characterize the binding effectivity of the ligands. This is clearly supported by the analysis of the corresponding data and speciation profile for the [(η^6^-*p*-cym)Ru]^2+^–H_2_-AlaAlaha^+^ system. Here, pM = 9.72 (see Table 3) and [M] = 1.9·10^−10^ M, which would indicate the stoichiometric binding of the metal ion by the ligand, but when pM’ = 6.11 and [M] = 7.76·10^−7^ M there is a completely different picture: only ca. 20% of the metal ion is complexed by the ligand and almost 80% of the organoruthenium cation exists in the form of hydroxido complexes.

Table 3 also shows the calculated pM’_1:1_ values because, in the case of the systems discussed here, if one of the synthesized metal–ligand complexes is dissolved, the equimolar condition (a 1:1 ratio of the components) is much more adequate than the 1:10 metal ion-to-ligand ratio corresponding to pM’.

**Figure 5:**
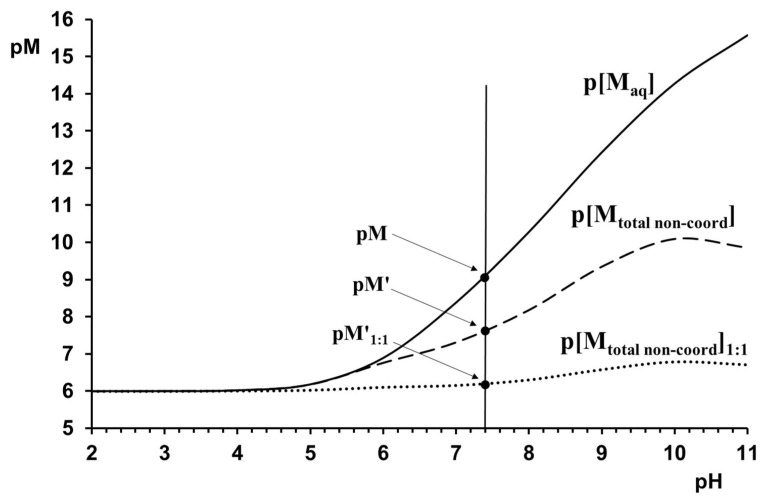
Dependence on the pH of p[M_aq_] or p[M_total non-coord_] at 1:10 metal ion-to-ligand ratio and p[M_total non-coord_]_1:1_ at 1:1 metal ion-to-ligand ratio calculated for the [(η^6^-*p*-cym)Ru(H_2_O)_3_]^2+^–H_2_-γ-Abha^+^ system (c_M_ = 10^−6^ M, I = 0.20 M KCl, t = 25 °C). (The pM, pM’ as well as pM’_1:1_ values can be read from the curves at pH = 7.4).

It is also worth mentioning that although pM, pM’, and pM’_1:1_ are important parameters to evaluate the metal-binding properties of ligands, their application is possible under several circumstances (e.g.*,* pH) only, under which they are calculated. To demonstrate this, the significant pH-dependence of the concentration of the free metal ion (p[M_aq_]), the concentration of the metal ion that is non-bonded by the ligand at 1:10 (p[M_total non-coord_]), as well as 1:1 (p[M_total non-coord_]_1:1_) metal ion-to-ligand ratios for the [(η^6^-*p*-cym)Ru(H_2_O)_3_]^2+^–H_2_-γ-Abha^+^ system are presented in Figure 5.

#### 3.1.3. A Comparison between the Metal Binding Effectivity of the Discussed Amino/Imidazole/Peptide-Hydroxamic Acids and Some Bidentate Bioactive Ligands Used to Develop Potential Drug Candidates

As is written in the Introduction and shown in numerous recent papers and reviews, such as references [58,60,61,77], and [97,98,99,100,101,102,103,104], among others, there is a great interest toward the development of new complexes with platinum group metals, including those with half-sandwich type organometallic cations. Predominantly, bidentate chelators have been involved to create new potential therapeutic agents with improved efficacy and selectivity. Despite the numerous works in this subject, only a few of them discuss the solution equilibrium results, especially under comparable conditions. For example, despite the fact that en-containing complexes with half-sandwich type organometallic Ru(II) cations have been known of since the beginning of this century [105,106], only a limited number of solution equilibrium results have been published on this system (e.g., any relevant data for the complexes in the presence of the competitor chloride ions has not been published [93]). Besides the previously discussed difficulties (kinetic inertness and strong competition with the hydrolytic processes of the organometallic cations), in some cases the too high stability of the metal complexes compared to the basicity of the chelating donors, e.g., with phen, bpy, and thiomaltol, allowed the estimation of the lower limit of the stability constants only [101,103]. Still, a comparison based on the collected results might be useful to make. The data, obtained for [(η^6^-*p*-cym)Ru(H_2_O)_3_]^2+^ and [(η^5^-Cp*)Rh(H_2_O)_3_]^2+^ complexes with some relevant (N,N), (N,O), (O,O), (S,O) donor-chelating ligands under comparable conditions to the ones used during the determination of the data in Table 1, have been used to calculate pM’1:1 values and are summarized in Table 4.

The pM’_1:1_ values in Table 4, and the corresponding values in Table 1, are used to make a comparison between the metal-binding effectivity of the various bidentate (N,N), (N,O), (O,O), and (S,O) chelators and the binding effectivity of the amino/imidazole/peptide hydroxamate derivatives at pH = 7.4 without any ligand excess at c_M_ = c_L_ = 1 × 10^−6^ M total concentrations in the presence or absence of the competitor chloride anions. The following conclusions can be drawn:

(1) Table 4 shows that, although the situation with dhp is a bit of an exception, it is true for all of the (O,O)-chelators (being either hydroxamate or maltolate derivatives) that, under the given conditions, these ligands are not able to bind these organometallic cations effectively. Rather, they exist at a high extent in the dissociated form in both the [(η^6^-*p*-cym)Ru(H_2_O)_3_]^2+^- and [(η^5^-Cp*)Rh(H_2_O)_3_]^2+^-containing systems. In agreement with this conclusion, the low binding effectivities of H_2_-γ-Abha^+^ and the dipeptide derivatives (see Table 1) provide additional support to the preference of the hydroxamate chelation with these ligands.

(2) Considering the various 5-membered (N,N) donors, the corresponding pM’_1:1_ values in Table 4 show that, although the hydroxide ions are strong competitors of the aliphatic en—especially in the [(η^6^-*p*-cym)Ru(H_2_O)_3_]^2+^ -containing system—all of the (N,N) donor ligands are much more effective chelators of the two organometallic cations than the abovementioned (O,O) ones. As is expectable, the 6-membered (N,N)-donor pn is significantly less efficient compared to en. However, out of the amino-hydroxamic acids, the effectivity of H_2_-β-Alaha^+^ to bind [(η^5^-Cp*)Rh(H_2_O)_3_]^2+^ is much higher than that of the 6-membered (N,N) donor pn; indeed, it is comparable with a 5-membered (N,N) ligand. There is no doubt that the most effective chelators of hydroxamic acid derivatives are the H_2_-α-Alaha^+^ and the imidazole-containing molecules. The effectivity of H_2_-α-Alaha^+^ in the absence of chloride anions, and that of the imidazole derivatives even in presence of chlorides, are close to the effectiveness of the (N,O)-donor 8-HQ, which, together with the (S,O)-donor thiomaltol, shows outstanding metal-binding ability towards the two organometallic cations. Although the efficiency of H_2_-α-Alaha^+^ and the imidazole-derivatives is perhaps a little less, their advantage is that they are capable of accepting more than one metal ion, which might allow the development of novel special multifunctional drug candidates.

### 3.2. Evaluation of the Stability Data for the Pd(II) Complexes with Several Peptidehydroxamates

It is well-known that Pt(II) and Pd(II), since they are typical soft metal ions, prefer to bind to soft N-donor ligands; even simple dipeptides bind these metal ions very effectively. [89]. Although the complex formation of these metal ions with hydroxamic acid derivatives of amino acids and peptides was previously studied in a few works, they provided very interesting results [54,55]. As is detailed in the Introduction, the only previous study on the interaction between Pt(II) and glycinehydroxamic acid revealed that Pt(II)-assisted hydrolysis of the coordinated ligand led to the formation of hydroxylamine and a N-bonded glycine complex under acidic conditions [54].

The high interest nowadays towards both the hydroxamate-based compounds and platinum metal-based complexes as potential chemotherapeutic candidates, as well as the interest in the development of multifunctional drug candidates by combining bioactive ligands and metal ions in the same complex [62], initiated research in our laboratory in this subject during the past years. In one work, the formation of stable ternary complexes of a peptide hydroxamic acid was described using [Pd(en)(H_2_O)_2_]^2+^ as a metal ion source [97]. In another ongoing work, the hydrolysis of the coordinated ligand was also observed in the interaction of [Pd(en)(H_2_O)_2_]^2+^ with H_2_-α-Alaha^+^ and H_2_-β-Alaha^+^ (unpublished results). The results to be discussed below come from the first—and, to the best of our knowledge, the only—solution study with [PdCl_4_]^2−^ as a metal ion source that relates to the interaction of Pd(II) with the primary and secondary di- and tripeptide hydroxamic acids: H_2_-AlaAlaha^+^, H_2_-NMeAlaAlaha^+^, H_2_-AlaGlyGlyha^+^, and H_2_-NMeAlaGlyGlyha^+^ [74]. The pH-potentiometric results, together with pM, pM’, pM’_1:1_, are summarized in Table 5.

**Figure 6:**
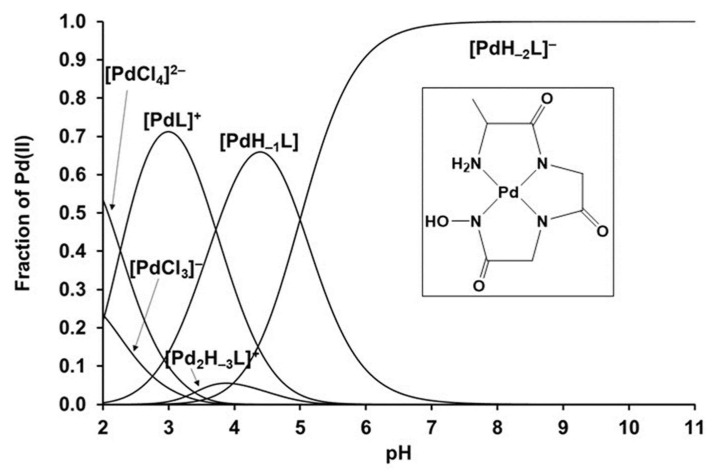
Concentration distribution curves calculated for the Pd(II)–H_2_-AlaGlyGlyha^+^ system at a 1:1 ratio and the suggested structure of [PdH_−2_L]^−^ (c_lig_ = 1.0 mM).

Table 5 indicates large differences among these four peptide hydroxamic acids in the extent of their interaction with Pd(II) (e.g., there are no results presented with H_2_-NMe-AlaAlaha^+^). The largest difference is whether there is only a reversible coordinative interaction between the metal ion and a certain ligand or are irreversible processes also in progress. Out of the four ligands studied, the complexation was found to be reversible only with H_2_-AlaGlyGlyha^+^, which is capable of completely taking the four coordination sites of Pd(II) at a 1:1 metal ion-to-ligand ratio, leading to the formation of a 4N coordinated mononuclear [PdH_−2_L]^−^. This (N_amino_,N_amide_,N_amide_,N_hydr_) donor set provides high stability over a wide pH-range; hydrolysis of the metal ion was not observed even under basic conditions (see Figure 6). Furthermore, due to the presence of a non-coordinated (O,O)-chelating function in [PdH_−2_L]^−^, following the deprotonation of the hydroxamate −OH, this ligand is capable of binding the metal ion excess, thereby resulting in the formation of a dinuclear species.

In contrast to H_2_-AlaGlyGlyha^+^, irreversible processes identified as the hydrolysis of the coordinated ligand were detected with the other three ligands under acidic conditions. Detailed ^1^H NMR studies have shown that there is a large difference in the rate of hydrolysis for the three ligands. While with H_2_-NMeAlaAlaha^+^ (which is capable of coordinating with a maximum of two nitrogens, (N_amino_,N_amide_) to a Pd(II) ion) the rather fast hydrolysis of the coordinated ligand together with the subsequent formation of elementary palladium hindered the equilibrium study, the hydrolytic processes were much slower with H_2_-AlaAlaha^+^ and H_2_-NMe-AlaGlyGlyha^+^.

The primary dipeptide derivative, H_2_-AlaAlaha^+^ (providing an (N_amino_,N_amide_,N_hydr_) donor set), was found to form Pd(II) complexes with the highest stability. At the same time, re-acidification and repeated titration of a a 1:1 Pd(II)–H_2_-AlaAlaha^+^ sample after four days revealed the disappearance of one equivalent of the base consumption in the titration curves compared to the fresh one, indicating that the hydrolysis of the ligand to the protonated hydroxylamine and the corresponding dipeptide was practically completed within this period of time. A similar process was also found with H_2_-NMe-AlaGlyGlyha^+^ (being capable of providing three nitrogens to form an (N_amino_,N_amide_,N_amide_) donor set). Here, new signals were already detected in the ^1^H NMR spectra of the acidic samples on one day but monitoring the samples for nine days showed a continuous increase of the amount of the hydrolyzed and coordinated ligand.

Although the rate of the Pd(II)-assisted hydrolysis of the latter two ligands shows some difference, this process is rather slow and requires several days in both systems. Consequently, speciation studies could successfully be performed on these systems (see Table 5).

Given that H_2_-AlaAlaha^+^ and H_2_-NMe-AlaGlyGlyha^+^ provide a maximum of three nitrogens for coordination with the (N_amino_,N_amide_,N_hydr_) and (N_amino_,N_amide_,N_amide_) donor sets, respectively, saturation of the fourth coordination site of the metal ion can be achieved by the coordination of an additional donor. This can be the deprotonated hydroxamate O with H_2_-NMe-AlaGlyGlyha^+^, while, due to steric reasons, it is not possible with H_2_-AlaAlaha^+^. This might be the reason behind the different composition of complexes formed with these ligands (Table 5). Namely, the tripeptide derivative cannot bind an excess of metal ion while the formation of bis-complexes and a trinuclear species have been described with H_2_-AlaAlaha^+^.

In terms of the Pd(II)-binding effectivity of these ligands, the pM values are worth evaluating.

With all three derivatives (although there are differences between the corresponding values) the data in Table 4 reveal a very high Pd(II)-binding ability (even if the competitive processes with chloride ions are taken into account (pM’)). Moreover, even at a 1:1 metal-to-ligand ratio at pH = 7.4, the stoichiometric amount of the Pd(II) is complexed by the peptide hydroxamic acids (see pM’_1:1_ values).

## 4. Conclusions

Since the complexes of platinum group metals with hydroxamic acid derivatives of amino acids, peptides, or analogous compounds of all the constituents themselves exhibit cytotoxic activity, they can be interesting candidates to develop multifunctional drug molecules with desired activities. For this work, knowledge of the stoichiometry and binding modes of such metal complexes, their thermodynamic parameters, and speciation profiles in solution can provide crucial information. However, due to the kinetic inertness of the complexes formed with these metal ions and the strong competitive hydrolytic processes, gaining such information is very difficult. In this review, the solution equilibrium results that were obtained so far in our laboratory on the complexes of Pd(II) and two other platinum group metal ions, the half-sandwich type [(η^6^-*p*-cym)Ru(H_2_O)_3_]^2+^ and [(η^5^-Cp*)Rh(H_2_O)_3_]^2+^, with hydroxamic acid derivatives of three amino acids, two imidazole analogous compounds, and four small peptides are summarized and evaluated. The main conclusions are as follows:

Out of the two discussed organometallic cations, [(η^6^-*p*-cym)Ru(H_2_O)_3_]^2+^ and [(η^5^-Cp*)Rh(H_2_O)_3_]^2+^, the former one, in a few systems, had its solution equilibrium measurements fail, either because the processes were too slow or the complexes formed were not water soluble.

All the studied hydroxamic acid derivatives can be very effective ligands of these organometallic cations. However, in a comparison, significant differences could be found among the complexes formed with the two metal ions and among the complexes formed with the different ligands regarding the stoichiometry, bonding modes, and the effectivity of the metal ion binding.

Unlike the three coordination sites offered by the organometallic cations for accepting donors, the relatively high number of donor atoms as well as their versatility in their position within the discussed ligands, in most cases, provides large diversity of coordination modes together with the frequent formation of oligonuclear species.

The coordination modes that are dominating in the complexes formed under certain conditions, as well as being either mono- or oligonuclear, are highly determined by the relative order of the pH-dependent conditional stability of the 5-membered hydroxamate-type (O,O) chelate and the other chelate(s), which may also be formed with a given ambidentate chelator ligand. In the case of the amino acid derivatives over a wide pH-range, the following stability order was found: 5-membered (N_amino_,N_hydr_) > 6-membered (N_amino_,N_hydr_)~5-membered hydroxamate-(O,O) >> 7-membered (N_amino_,N_hydr_). Consequently, with the α-derivative, the (O,O) chelate, except the highly acidic pH-range, can only coordinate at metal ion excess. With the β one, a dinuclear complex predominates even at a 1:1 metal-to-ligand ratio, while with the γ-derivative, in the preferred coordination mode, the (O,O) chelate and the γ-amino-N, being part of another complex/ligand, saturate the three sites of both organometallic cations.

With the imidazole derivatives the imidazole-N(3) is the main anchor and forms a chelate with the hydroxamate-N in the complexes of the primary H_2_-Imcha^+^ and the hydroxamate-O for the secondary derivative, H_2_-NMe-Imcha^+^. The stability order in the whole measured pH-range is: 5-membered (N_im_,N_hydr_) > 6-membered (N_im_,O_hydr_) > 5-membered hydroxamate-(O,O). Moreover, with the imidazole derivatives, interesting oligonuclear complexes are formed, in which the third coordination site of each organometallic cation is occupied by an imidazole-N(1).

In the complexes with all four of the studied peptide hydroxamic acids, the 5-membered hydroxamate-(O,O) (for the primary derivatives, at higher pH, the doubly deprotonated hydroxymate-(O,O)) is the predominant chelate. With primary derivatives, Nitrogen can only coordinate to a second metal ion. At higher pH, with the secondary H_2_-NMeAlaAlaha^+^, the effect of the different hydrolytic activities of the two organometallic cations on the metal–ligand interaction is also well demonstrated.

Although there are differences in the binding capabilities of the discussed ligands based on the thermodynamic stability constants of their complexes, all of them could be very effective chelators for binding [(η^5^-Cp*)Rh(H_2_O)_3_]^2+^ and especially [(η^6^-p-cym)Ru(H_2_O)_3_]^2+^ (H_2_-γ-Abha^+^ might be the only exception in presence of Cl^−^ anions), as it is clearly shown by the pM values in Table 3. At the same time, since the hydrolytic processes of these cations (especially for the organoruthenium) are very strong competitors of the metal–ligand interactions, the binding ability of some of these ligands can decrease significantly, even at a 1:10 metal ion-to-ligand ratio (pM’) and especially without ligand excess (pM’_1:1_).

In the Pd(II)-peptide hydroxamic acid systems, a high preference toward N-donor chelation over the (O,O) one was revealed. With the exception of the H_2_-AlaGlyGlyha^+^–containing system, in which an (N_amino_,N_amide_,N_amide_,N_hydr_) donor set saturates the coordination sphere of Pd(II), complex formation Pd(II)-assisted hydrolysis of the coordinated ligand was also detected. However, the rate of the hydrolysis of the coordinated ligand can be controlled and fine-tuned through proper selection of the number and type of the coordinated N-donors.

Taken together, all the information summarized in the present review may provide a significant contribution to the design and synthesis of platinum metal complexes to develop effective drug candidate compounds.

## Figures and Tables

**Table 1:** Dissociation macro constants (p*K*_i_) and micro constants (p*K*_i_^micro^) of some selected amino and peptidehydroxamic acids at 25.0 °C (I = 0.20 M KCl).

	H_2_-α-Alaha^+^ [82]	H_2_-β-Alaha^+^ [82]	H_2_-γ-Abha^+^ [83]	H_2_-AlaAlaha^+^ [37]
p*K*_1_	7.34	8.32	8.76	7.66
p*K*_2_	9.16	9.59	10.19	8.88
*K*_1_^micro^/*K*_2_^micro^	2.70	0.42	0.14	8.32
p*K*_1_^micro^	7.48	8.85	9.67	7.71
p*K*_2_^micro^	7.90	8.47	8.82	8.63
p*K*_3_^micro^	9.02	9.06	9.28	8.83
p*K*_4_^micro^	8.60	9.44	10.13	7.91

**Table 2:** Thermodynamic (log*β*) and conditional stability constants (logβ*) of [(η^6^-*p*-cym)Ru(H_2_O)_3_]^2+^ and [(η^5^-Cp*)Rh(H_2_O)_3_]^2+^ hydroxido complexes at 25.0 °C and I = 0.20 M KNO_3_ and KCl, respectively.

	M = [(η^6^*-p*-cym)Ru] [84]		M = [(η^5^-Cp*)Rh] [85]	
	0.20 M KNO_3_	0.20 M KCl	0.20 M KNO_3_	0.20 M KCl
[M_2_(OH)_2_]^2+^	−	−7.12	−8.53	−11.12
[M_2_(OH)_3_]^+^	−9.16	−11.88	−14.26	−19.01

**Table 3:** Dissociation constants (p*K*_a_) of the ligands and overall stability constants (log*β*) ^a^ for their [(η^6^-*p*-cym)Ru]^2+^ and [(η^5^-Cp*)Rh]^2+^ complexes determined at t = 25 °C, I = 0.20 M KCl (values in bold) or KNO_3_, as well as the derived constants: pM, pM’, and pM’_1:1_.

		Species	[(η^6^-*p*-cym)Ru]^2+^	[(η^5^-Cp*)Rh]^2+^	
Refs. [52,53]	H_2_-α-Alaha^+^ p*K*_1_ = 7.38 p*K*_2_ = 9.20	[MHL]^2+^	**15.86**	−	
		[ML]^+^	**11.83**	14.78	
		[MH_−1_L]	**2.42**	5.20	
		[M_2_H_−1_L]^2+^	**12.38**	15.27	
		[M_2_H_−2_L]^+^	**3.1**	6.06	
		[M_2_H_−3_L]	**−**	−5.09	
		pM	10.69	13.64	
		pM’	10.43	13.64	
		pM’_1:1_	7.28	10.29	
	H_2_-β-Alaha^+^ p*K*_1_ = 8.43 p*K*_2_ = 9.77	[MHL]^2+^	**17.99**	17.71	
		[ML]^+^	**12.75**	−	
		[MH_−1_L]	**2.54**	−	
		[M_2_L_2_]^2+^	**−**	29.44	
		[M_2_H_−1_L_2_]^+^	**−**	21.88	
		[M_2_H_−1_L]^2+^	**11.83**	14.16	
		[M_2_H_−2_L]^+^	**−**	4.79	
		pM	10.27	9.78	
		pM’	9.76	9.77	
		pM’_1:1_	6.74	9.00	
	H_2_-γ-Abha^+^ p*K*_1_ = 8.79 p*K*_2_ = 10.24	[MHL]^2+^	**18.71**	18.15	**16.66**
		[ML]^+^	**12.34**	−	**−**
		[MH_−1_L]	**3.74**	−	**−**
		[M_2_L_2_]^2+^	**−**	27.10	**22.88**
		[M_2_H_−1_L_2_]^+^	**−**	19.44	**14.82**
		[M_2_H_−1_L]^2+^	**11.45**	11.34	**7.66**
		[M_2_H_−2_L]^+^	**3.50**	−	**−**
		pM	9.12	8.00	6.40
		pM’	7.61	7.56	6.40
		pM’_1:1_	6.20	6.50	6.07
Ref. [91]	H_2_-Imcha^+^ **p*K*_1_ = 3.73** **p*K*_2_ = 8.85**	[MHL]^2+^	**precipitation over a wide pH-range**	**14.53**	
		[ML]^+^		**12.63**	
		[MH_−1_L]		**6.56**	
		[M_2_H_−1_L]^2+^		**10.92**	
		[M_2_H_−2_L]^+^		**5.06**	
		pM	−	13.47	
		pM’		13.47	
		pM’_1:1_		9.39	
	H_2_-NMe-Imcha^+^ **p*K*_1_ = 4.83** **p*K*_2_ = 8.20**	[MHL]^2+^	**15.5**	**15.37**	
		[ML]^+^	**11.8**	**10.82**	
		[MH_−1_L]	**5.3**	**4.84**	
		pM	12.84	12.35	
		pM’	12.84	12.35	
		pM’_1:1_	8.37	8.40	
Ref. [75]	H_2_-AlaAlaha^+^ p*K*_1_ = 7.77 p*K*_2_ = 8.89	[MHL]^2+^	16.09	15.54	
		[M_2_L]^3+^	14.95	13.57	
		[M_2_H_−1_L]^2+^	11.16	10.07	
		[M_2_H_−2_L]^+^	4.63	3.40	
		[M_2_H_−3_L]^2+^	−4.79	−5.47	
		pM	9.72	8.78	
		pM’	6.11	8.66	
		pM’_1:1_	6.01	7.78	
	H_2_-NMe-AlaAlaha^+^ p*K*_1_ = 7.77 p*K*_2_ = 8.70	[MHL]^2+^	17.53	15.89	
		[ML]^+^	11.61	9.48	
		[MH_−1_L]	2.50	2.62	
		[M_2_L]^3+^	−	14.16	
		pM	10.76	9.26	
		pM’	8.18	9.22	
		pM’_1:1_	6.29	6.82	
	H_2_-AlaGlyGlyha^+^ p*K*_1_ = 7.74 p*K*_2_ = 8.82	[MHL]^2+^	17.19	15.35	
		[ML]^+^	13.33	10.42	
		[MH_−1_L]	slow equilibrium processes at pH > 6	3.42	
		[MH_−2_L]^−^		−6.91	
		[M_2_L]^3+^		13.6	
		[M_2_H_−1_L]^2+^		9.53	
		[M_2_H_−2_L]^+^		4.12	
		pM	−	10.00	
		pM’		9.99	
		pM’_1:1_		8.31	
	H_2_-NMe-AlaGlyGlyha^+^ p*K*_1_ = 7.69 p*K*_2_ = 8.57	[MHL]^2+^	17.14	15.53	
		[ML]^+^	slow equilibrium processes at pH > 6	10.56	
		[MH_−1_L]		2.42	
		[MH_−2_L]^−^		−7.07	
		[M_2_L]^3+^		14.0	
		[M_2_H_−1_L]^2+^		9.62	
		pM	−	9.94	
		pM’		9.63	
		pM’_1:1_		7.37	

^a^ With Ru(II) and Rh(III), their organometallic arene or arenyl half-sandwich type cations were defined as M. Component H, in general, refers to a dissociable proton, H_−1_, which represents either metal-induced deprotonation of the primary derivatives (R_N_ = H) (formation of the doubly deprotonated hydroximato ligand), deprotonation of the peptide bond or imidazole-NH, or coordination of a hydroxide ion. The data in bold refers to that data obtained at an chloride medium.

**Table 4:** pM’_1:1_ values (c_M_ = c_L_ = 1·10^−6^ M, pH = 7.4) calculated for the solutions containing [(η^6^-*p*-cym)Ru(H_2_O)_3_]^2+^ or [(η^5^-Cp*)Rh(H_2_O)_3_]^2+^, and one of the selected bidentate (N,N), (N,O, (O,O), and (S,O) chelators in presence or absence of chloride anion as a competitor ligand.

I = 0.20 M KNO_3_			I = 0.20 M KCl		
	Ru^2+^	Rh^2+^		Ru^2+^	Rh^2+^
en 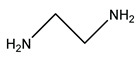	7.61 [101]	9.04 [101]	en 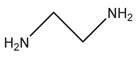	—	9.70 [77]
pn 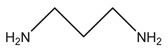	—	7.16 [53]	dhp 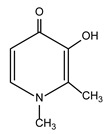	6.82 [98]	6.39 [100]
phen 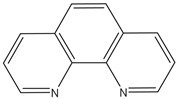	>8 [101]	8.00 [101]	maltol 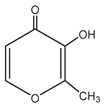	6.14 [98]	6.12 [85]
bpy 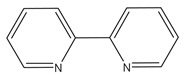	>8 [101]	8.00 [101]	allomaltol 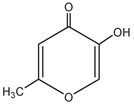	6.08 [99]	6.13 [85]
8-HQ 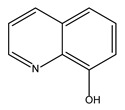	11.47 [102]	10.91 [102]	ethyl maltol 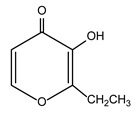	6.11 [99]	—
thiomaltol 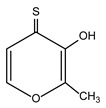	—	>12 [103]	thiomaltol 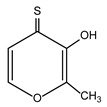	>9 [99]	>12 [103]
acetohydroxamic acid 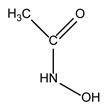	6.03 [104]	6.15 [104]	thiodhp 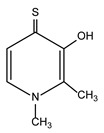	>8 [99]	—
*N*-methyl-acetohydroxamic acid 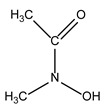	6.25 [104]	6.24 [104]	*N*-methyl-acetohydroxamic acid * 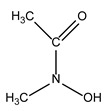 *	6.31 [98]	—

**Table 5:** Dissociation constants (p*K*) of the ligands and overall stability constants (log*β*) of their Pd(II) complexes at I_tot_ = 0.20 M (0.10 M KCl + 0.10 M KNO_3_) and 25.0 °C.

	H_2_-AlaAlaha^+^	H_2_-AlaGlyGlyha^+^	H_2_-NMeAlaGlyGlyha^+^
	p*K*_1_ = 7.70 p*K*_2_ = 8.80	p*K*_1_ = 7.70 p*K*_2_ = 8.80	p*K*_1_ = 7.78 p*K*_2_ = 8.74
	[74]		
[PdL]^+^	22.84	22.70	22.60
[PdH_−1_L]	20.52	18.98	18.69
[PdH_−2_L]^−^	11.5	13.98	12.2
[Pd_2_H_−3_L]	−	22.96	−
[Pd_2_H_−4_L]^−^	−	13.9	−
[Pd_3_H_−4_L_2_]	44.4	−	−
[PdL_2_]	33.4	−	−
[PdH_−1_L_2_]^−^	24.8	−	−
pM	27.05	27.88	26.13
pM’	19.35	20.19	18.43
pM’_1:1_	16.38	17.23	15.47

## Data Availability

Data reviewed in this work were taken from the cited references.

## Abbreviations

Aha^−^	acetohydroxamate
AlaAlaha^−^	alanyl-alanine hydroxamate
AlaGlyGlyha^−^	alanyl-glycyl-glycine hydroxamate
bpy	2,2′-bipyridine
c_M_	total concentration of the metal ion
Cp*	pentamethyl-cyclopentadienyl anion
DFT	Density Functional Theory
dien	diethylenetriamine
en	ethylenediamine
ESI-TOF-MS	Electrospray Ionization Time-of-flight Mass Spectrometry
GlyGlyAla	glycyl-glycyl-alanine
Imcha^−^	imidazole-carbohydroxamate
L	fully deprotonated form of a certain ligand
MCs	metallacrowns
NMe-AlaAlaha^−^	*N*-methyl-alanyl-alanine hydroxamate
NMe-AlaGlyGlyha^−^	*N*-methyl-alanyl-glycyl-glycine hydroxamate
NMe-Imcha^−^	*N*-methyl-imidazole-carbohydroxamate
NMR	Nuclear Magnetic Resonance Spectroscopy
*p*-cym	*p*-cymene (1-methyl-4-isopropylbenzene)
pic	2-picolylamine
pM	negative logarithm of the concentration of the free metal ion; c_M_ 10^−6^ M, M:L = 1:10
pM’	negative logarithm of the total concentration of the non-chelatedmetal ion; cM = 10−6 M, M:L= 1:10
pn	propylenediamine
terpy	terpyridine
UV-VIS	Ultraviolet-visible Spectroscopy
α-Alaha^−^	α-alaninehydroxamate
*β**/*K*’	conditional overall/stepwise stability constant, in which the extent of the competing processes under a certain condition is quantified. It is valid only under the given experimental condition
β-Alaha^−^	β-alaninehydroxamate
γ-Alaha^−^ (GABAha^−^)	γ-alaninehydroxamate

## References

[1] Chatterjee B. (1978). Donor properties of hydroxamic acids. Coord. Chem. Rev..

[2] Agrawal Y.K. (1979). Hydroxamic Acids and Their Metal Complexes. Russian Chem. Rev..

[3] Raymond K.N., Müller G., Matzanke B.F. (1984). Complexation of iron by siderophores. A review of their solution and structural chemistry and biological function. Topics in Current Chemistry.

[4] Hancock R.D., Martell A.E. (1989). Ligand Design for Selective Complexation of Metal Ions in Aqueous Solution. Chem. Rev..

[5] Crumbliss A. (1990). Iron bioavailability and the coordination chemistry of hydroxamic acids. Coord. Chem. Rev..

[6] Albrecht-Gary A.M., Crumbliss A.L., Sigel A., Sigel H. (1998). Coordination chemistry of siderophores: Thermodynamics and kinetics of iron chelation and release. Metal Ions in Biological Systems.

[7] Kiss T., Farkas E. (1998). Metal-binding ability of desferrioxamine B. J. Incl. Phenom. Mol. Recogn. Chem..

[8] Whittaker M., Floyd C.D., Brown P., Gearing A.J.H. (1999). Design and Therapeutic Application of Matrix Metalloproteinase Inhibitors. Chem. Rev..

[9] Muri E.M.F., Nieto M.J., Sindelar R.D., Williamson J.S. (2002). Hydroxamic Acids as Pharmacological Agents. Current Med. Chem..

[10] Liu Z.D., Hider R.C. (2002). Design of iron chelators with therapeutic application. Coord. Chem. Rev..

[11] Raymond K.N., Dertz E.A., Crosa J.H., Mey A.R., Payne S.M. (2004). Biochemical and physical properties of siderophores. Iron Transport in Bacteria.

[12] Marmion C.J., Griffith D., Nolan K.B. (2004). Hydroxamic Acids—An Intriguing Family of Enzyme Inhibitors and Biomedical Ligands. Eur. J. Inorg. Chem..

[13] Codd R. (2008). Traversing the coordination chemistry and chemical biology of hydroxamic acids. Coord. Chem. Rev..

[14] Griffith D., Devocelle M., Marmion C.J., Hughes A. (2009). Hydroxamic acids: Their chemistry, bioactivity, solution and solid phase synthesis. Amino Acids, Peptides and Proteins in Organic Chemistry.

[15] Li Z., Yamamoto H. (2012). Hydroxamic Acids in Asymmetric Synthesis. Acc. Chem. Res..

[16] Gupta S.P. (2013). Hydroxamic Acids: A Unique Family of Chemicals with Multiple Biological Activities.

[17] Natarajan R., Gupta S. (2013). Hydroxamic Acids as Chelating Mineral Collectors. Hydroxamic Acids.

[18] Marmion C.J., Parker J.P., Nolan K.B. (2013). Hydroxamic Acids: An Important Class of Metalloenzyme Inhibitors. Compr. Inorg. Chem..

[19] Bertrand S., Hélesbeux J.-J., Larcher G., Duval O. (2013). Hydroxamate, a Key Pharmacophore Exhibiting a Wide Range of Biological Activities. Mini Rev. Med. Chem..

[20] Osipov V.N., Khachatryan D.S., Balaev A.N. (2020). Biologically active quinazoline-based hydroxamic acids. Med. Chem. Res..

[21] Bíró L., Buglyó P., Farkas E. (2021). Factors Determining the Metal Ion Binding Ability and Selectivity of Hydroxamate-Based Compounds. Curr. Med. Chem..

[22] Wang Z., Wang L., Fan R., Zhou J., Zhong J. (2016). Molecular design and structural optimization of potent peptide hydroxamate inhibitors to selectively target human ADAM metallopeptidase domain 17. Comput. Biol. Chem..

[23] Song W.Q., Liu M.L., Li S.Y., Xiao Z.P. (2021). Recent Efforts in the Discovery of Urease Inhibitor Identifications. Curr. Top Med. Chem..

[24] Moreno-Yruela C., Bæk M., Vrsanova A.E., Schulte C., Maric H.M., Olsen C.A. (2021). Hydroxamic acid-modified peptide microarrays for profiling isozyme-selective and inhibition of histone deacetylases. Nat. Commun..

[25] Mahindra A., Millard C.J., Black I., Archibald L.J., Schwabe J.W.R., Jamieson A.G. (2019). Synthesis of HDAC Substrate Peptidomimetic Inhibitors Using Fmoc Amino Acids Incorporating Zinc-Binding Groups. Org. Lett..

[26] Crisponi G., Remelli M. (2008). Iron chelating agents for the treatment of iron overload. Coord. Chem. Rev..

[27] Szebesczyk A., Olshvang E., Shanzer A., Carver P.L., Gumienna-Kontecka E. (2016). Harnessing the power of fungal siderophores for the imaging and treatment of human diseases. Coord. Chem. Rev..

[28] Schalk I.J. (2018). Siderophore–antibiotic conjugates: Exploiting iron uptake to deliver drugs into bacteria. Clin. Microbiol. Infect..

[29] Apfel C., Banner D.W., Bur D., Dietz M., Hirata T., Hubschwerlen C., Locher H., Page M.G.P., Pirson W., Rosse G. (2000). Hydroxamic Acid Derivatives as Potent Peptide Deformylase Inhibitors and Antibacterial Agents. J. Med. Chem..

[30] Pavelic S.K., Sedic M., Poznic M., Rajic Z., Zorc B., Pavelic K., Balzarini J., Mintas M. (2010). Evaluation of in vitro biological activity of O-alkylated hydroxamic derivatives of some nonsteroidal antiinflammatory drugs. Anticancer Res..

[31] Neganova M.E., Klochkov S.G., Aleksandrova Y.R., Aliev G. (2021). The Hydroxamic Acids as a Potential Anticancer and Neuroprotective Agents. Curr. Med. Chem..

[32] Cross J.M., Blower T.R., Kingdon A.D.H., Pal R., Picton D.M., Walton J.W. (2020). Anticancer Ruthenium Complexes with HDAC Isoform Selectivity. Molecules.

[33] Hanif M., Arshad J., Astin J., Rana Z., Zafar A., Movassaghi S., Leung E., Patel K., Söhnel T., Reynisson J. (2020). A Multitargeted Approach in the Discovery of an Organorhodium Anticancer Agent Based On Vorinostat as a Potent Histone Deacetylase Inhibitor. Angew. Chem. Int. Ed..

[34] Kurzak B., Kozłowski H., Farkas E. (1992). Hydroxamic and aminohydroxamic acids and their complexes with metal ions. Coord. Chem. Rev..

[35] Enyedy É.A., Csóka H., Lázár I., Micera G., Garribba E., Farkas E. (2002). Effects of side chain amino nitrogen donor atoms on metal complexation of aminohydroxamic acids: New diaminohydroxamates chelating Ni(ii) more strongly than Fe(iii). J. Chem. Soc. Dalton Trans..

[36] Csapó E., Buglyó P., Nagy N.V., Santos M.A., Corona A., Farkas E. (2010). Synthesis and characterization of Cu^2+^, Ni^2+^ and Zn^2+^ binding capability of histidinehydroxamic acid derivatives. Polyhedron.

[37] Buglyó P., Nagy E.M., Farkas E., Sóvágó I., Sanna D., Micera G. (2007). New insights into the metal ion–peptide hydroxamate interactions: Metal complexes of primary hydroxamic acid derivatives of common dipeptides in aqueous solution. Polyhedron.

[38] Buglyó P., Nagy E.M., Sóvágó I., Ozsváth A., Sanna D., Farkas E. (2016). Metal ion binding capability of secondary (*N*-methyl) versus primary (N–H) dipeptide hydroxamic acids. Polyhedron.

[39] Farkas E., Csapó E., Buglyó P., Damante C.A., Natale G.D. (2009). Metal-binding ability of histidine-containing peptidehydroxamic acids: Imidazole versus hydroxamate coordination. Inorg. Chim. Acta..

[40] Cal M., Jaremko Ł., Jaremko M., Stefanowicz P. (2013). The metallacrowns as templates for spontaneous self-assembly of polypeptides into a tetrahelical bundle. New J. Chem..

[41] Cal M., Kotynia A., Jaremko L., Jaremko M., Lisowski M., Cebo M., Brasu J., Stefanowicz P. (2015). Metallacrowns as products of the aqueous medium self-assembly of histidinehydroxamic acid containing polypeptides. Dalton Trans..

[42] Bodwin J.J., Cutland A.D., Malkani R.G., Pecoraro V.L. (2001). The development of chiral metallacrowns into anion recognition agents and porous materials. Coord. Chem. Rev..

[43] Mezei G., Zaleski C.M., Pecoraro V.L. (2007). Structural and functional evolution of metallacrowns. Chem. Rev..

[44] Zhao X.J., Zhang Q.F., Li D.C., Dou J.M., Wang D.Q. (2010). Syntheses, structural characterizations and properties of 12-MC-4 organotin(IV) metallacrowns: [12-MCRSn(IV)N(shi)-4] and [12-MCRSn(IV)N(Clshi)-4] (R = Et, Bu, Ph; H3shi = salicylhydroxamic acid; H3Clshi = 5-chlorosalicylhydroxamic acid. J. Organomet. Chem..

[45] Tegoni M., Remelli M. (2012). Metallacrowns of copper(II) and aminohydroxamic acids. Thermodynamics of self-assembly and host-guest equilibria. Coord. Chem. Rev..

[46] Chow C.Y., Trivedi E.R., Pecoraro V., Zaleski C.M. (2015). Heterometallic Mixed 3d-4f Metallacrowns: Structural Versatility, Luminescence, and Molecular Magnetism. Comments Inorg. Chem..

[47] Happ P., Plenk C., Rentschler E. (2015). 12-MC-4 metallacrowns as versatile tools for SMM research. Coord. Chem. Rev..

[48] Ostrowska M., Fritsky I.O., Gumienna-Kontecka E., Pavlishchuk A.V. (2016). Metallacrown-based compounds: Applications in catalysis, luminescence, molecular magnetism, and adsorption. Coord. Chem. Rev..

[49] Jankolovits J., Kampf J.W., Pecoraro V.L. (2015). Assembly of zinc metallacrowns with an α-amino hydroxamic acid ligand. Chin. Chem. Lett..

[50] Severin K. (2003). Self-assembled organometallic receptors for small ions. Coord. Chem. Rev..

[51] Kathó Á., Carmona D., Viguri F., Remacha C.D., Kovács J., Joó F., Oro L.A. (2000). Enantioselective hydride transfer hydrogenation of ketones catalyzed by [(η6-p-cymene)Ru(amino acidato)Cl] and [(η6-p-cymene)Ru(amino acidato)]3(BF4)3 complexes. J. Organomet. Chem..

[52] Parajdi-Losonczi P.L., Bényei A.C., Kováts É., Timári I., Muchova T.R., Novohradsky V., Kasparkova J., Buglyó P. (2016). [(η^6^-*p*-cymene)Ru(H_2_O)_3_]^2+^ binding capability of aminohydroxamates—A solution and solid state study. J. Inorg. Biochem..

[53] Parajdi-Losonczi P.L., Buglyó P., Skakalova H., Kasparkova J., Lihi N., Farkas E. (2018). Half-sandwich type rhodium(III)–aminohydroxamate complexes: The role of the position of the amino group in metal ion binding. New J. Chem..

[54] Davies H.O., Brown D.A., Yanovsky A.I., Nolan K.B. (1995). Synthesis of trans-bis (glycinehydroxamato) platinum (II) hydrate tans-dichlorobis (glycylglycine) platinum (II) dihydrate and the crystal and molecular structures of trans-dichlorobis (glycine) platinum (II) dihydrate and trans, trans-dichlorobis (glycinato) platinum (IV), hydrolysis products formed in reactions of the former complexes. Inorg. Chim. Acta.

[55] Griffith D., Lyssenko K., Jensen P., Kruger P.E., Marmion C.J. (2005). Novel platinum(ii) ammine hydroxamate and hydroximate complexes and the platinum-assisted hydrolysis of hydroxamic acids. Dalton Trans..

[56] Moore W.M., Spilburg C.A. (1986). Purification of human collagenases with a hydroxamic acid affinity column. Biochemistry.

[57] Jiang Z., You Q., Zhang X. (2019). Medicinal chemistry of metal chelating fragments in metalloenzyme active sites: A perspective. Eur. J. Med. Chem..

[58] Tremlett W.D.J., Goodman D.M., Steel T.R., Kumar S., Wieczorek-Błauż A., Walsh F.P., Sullivan M.P., Hanif M., Hartinger C.G. (2021). Design concepts of half-sandwich organoruthenium anticancer agents based on bidentate bioactive ligands. Coord. Chem. Rev..

[59] Ndagi U., Mhlongo N., Soliman M. (2017). Metal complexes in cancer therapy–an update from drug design perspective. Drug Des. Devel. Ther..

[60] Kitteringham E., McKeon A.M., O’Dowd P., Devocelle M., Murphy B.M., Griffith D.M. (2020). Synthesis and characterisation of a novel mono functionalisable Pt(IV) oxaliplatin-type complex and its peptide conjugate. Inorg. Chim. Acta.

[61] Barnard C. (2017). Platinum Group Metal Compounds in Cancer Chemotherapy. Johnson Matthey Technol. Rev..

[62] Kenny R.G., Marmion C.J. (2019). Toward Multi-Targeted Platinum and Ruthenium Drugs-A New Paradigm in Cancer Drug Treatment Regimens?. Chem. Rev..

[63] Ang W.H., Dyson P.J. (2006). Classical and Non-Classical Ruthenium-Based Anticancer Drugs: Towards Targeted Chemotherapy. Eur. J. Inorg. Chem..

[64] Meier-Menches S.M., Gerner C., Berger W., Hartinger C.G., Keppler B.K. (2018). Structure-Activity Relationships for Ruthenium and Osmium Anticancer Agents-Towards Clinical Development. Chem. Soc. Rev..

[65] Zeng L., Gupta P., Chen Y., Wang E., Ji L., Chao H., Chen Z.S. (2017). The Development of Anticancer Ruthenium(II) Complexes: From Single Molecule Compounds to Nanomaterials. Chem. Soc. Rev..

[66] Golbaghi G., Castonguay A. (2020). Rationally Designed Ruthenium Complexes for Breast Cancer Therapy. Molecules.

[67] Lin K., Zhao Z.-Z., Bo H.-B., Hao X.-J., Wang J.-Q. (2018). Applications of Ruthenium Complex in Tumor Diagnosis and Therapy. Front. Pharmacol..

[68] Gichumbi J.M., Friedrich H.B. (2018). Half-sandwich complexes of platinum group metals (Ir, Rh, Ru and Os) and some recent biological and catalytic applications. J. Organomet. Chem..

[69] Odularu A.T., Ajibade P.A., Mbese J.Z., Oyedeji O.O. (2019). Developments in Platinum-Group Metals as Dual Antibacterial and Anticancer Agents. J. Chem..

[70] Lee S.Y., Kim C.Y., Nam T.-G. (2020). Ruthenium Complexes as Anticancer Agents: A Brief History and Perspectives. Drug Des. Dev. Ther..

[71] Mészáros J.P., Dömötör O., Hackl C.M., Roller A., Keppler B.K., Kandioller W., Enyedy É.A. (2018). Structural and solution equilibrium studies on half-sandwich organorhodium complexes of (N,N) donor bidentate ligands. New J. Chem..

[72] Pivarcsik T., Dömötör O., Mészáros J.P., May N.V., Spengler G., Csuvik O., Szatmári I., Enyedy É.A. (2021). 8-Hydroxyquinoline-amino acid hybrids and their half-sandwich Rh and Ru complexes: Synthesis, anticancer activities, solution chemistry and interaction with biomolecules. Int. J. Mol. Sci..

[73] Dömötör O., Pivarcsik T., Mészáros J.P., Szatmári I., Fülöp F., Enyedy É.A. (2021). Critical factors affecting the albumin binding of half-sandwich Ru(II) and Rh(III) complexes of 8-hydroxyquinolines and oligopyridines. Dalton Trans..

[74] Ozsváth A., Farkas E., Diószegi R., Buglyó P. (2019). Versatility and trends in the interaction between Pd(II) and peptide hydroxamic acids. New J. Chem..

[75] Ozsváth A., Bíró L., Nagy E.M., Buglyó P., Sanna D., Farkas E. (2019). Trends and Exceptions in the Interaction of Hydroxamic Acid Derivatives of Common Di- and Tripeptides with Some 3d and 4d Metal Ions in Aqueous Solution. Molecules.

[76] Cotton F.A., Wilkinson G. (1988). Advanced Inorganic Chemistry.

[77] Peacock A.F.A., Habtemariam A., Fernández R., Walland V., Fabbiani F.P.A., Parsons S., Aird R.E., Jodrell D.I., Sadler P.J. (2006). Tuning the Reactivity of Osmium(II) and Ruthenium(II) Arene Complexes under Physiological Conditions. J. Am. Chem. Soc..

[78] Senent M.L., Niño A., Caro C.M., Ibeas S., García B., Leal J.M., Secco F., Venturini M. (2003). Deprotonation Sites of Acetohydroxamic Acid Isomers. A Theoretical and Experimental Study. J. Org. Chem..

[79] García B., Ibeas S., Leal J.M., Secco F., Venturini M., Senent M.L., Niño A., Muñoz C. (2005). Conformations, Protonation Sites, and Metal Complexation of Benzohydroxamic Acid. A Theoretical and Experimental Study. Inorg. Chem..

[80] Brink C.P., Crumbliss A.L. (1984). Kinetics, mechanism, and thermodynamics of aqueous iron(III) chelation and dissociation: Influence of carbon and nitrogen substituents in hydroxamic acid ligands. Inorg. Chem..

[81] Farkas E., Bátka D., Csapó E., Buglyó P., Haase W., Sanna D. (2007). Synthesis and characterization of Cu^2+^, Ni^2+^ and Zn^2+^ binding capability of some amino- and imidazole hydroxamic acids: Effects of substitution of side chain amino-N for imidazole-N or hydroxamic-N-H for -N-CH3 on metal complexation. Polyhedron.

[82] Farkas E., Kiss T., Kurzak B. (1990). Microscopic dissociation processes of alaninehydroxamic acids. J. Chem. Soc. Perkin Trans..

[83] Tegoni M., Ferretti L., Sansone F., Remelli M., Bertolasi V., Dallavalle F. (2007). Synthesis, solution thermodynamics, and X-ray study of CuII-[12]Metallacrown-4 with GABA Hydroxamic Acid: An Unprecedented Crystal Structure of a [12]MC-4 with a γ-Aminohydroxamate. Chem. Eur. J..

[84] Bíró L., Farkas E., Buglyó P. (2012). Hydrolytic behaviour and chloride ion binding capability of [(η^6^-*p*-cym)Ru(H_2_O)_3_]^2+^: A solution equilibrium study. Dalton Trans..

[85] Dömötör O., Aicher S., Schmidlehner M., Novak M.S., Roller A., Jakupec M.A., Kandioller W., Hartinger C.G., Keppler B.K., Enyedy É.A. (2014). Antitumor pentamethylcyclopentadienyl rhodium complexes of maltol and allomaltol: Synthesis, solution speciation and bioactivity. J. Inorg. Biochem..

[86] Bíró L., Godó A.J., Bihari Z., Garribba E., Buglyó P. (2013). Tuning the hydrolytic properties of half-sandwich-type organometallic cations in aqueous solution. Eur. J. Inorg. Chem..

[87] Wheate N.J., Walker S., Craig G.E., Oun R. (2010). The status of platinum anticancer drugs in the clinic and in clinical trials R. Dalton Trans..

[88] Baes C.F., Mesmer R.E. (1976). Palladium and platinum in the Hydrolysis of Cations.

[89] Ágoston C.G., Janowska T.K., Sóvágó I. (1999). Potentiometric and NMR studies on palladium(II) complexes of oligoglycines and related ligands with non-coordinating side chains. J. Chem. Soc. Dalton Trans..

[90] Elding L.I. (1972). Palladium(II) halide complexes. I. Stabilities and spectra of palladium(II) chloro and bromo aqua complexes. Inorg. Chim. Acta.

[91] Parajdi-Losonczi P.L. (2018). Hydroxamate-complexes of half-sandwich platinum metal ions: Solution equilibrium studies and synthesis. Ph.D. Thesis.

[92] Hassoon A.A., Szorcsik A., Bogár F., Papp I.Z., Fülöp L., Kele Z., Gajda T. (2021). The interaction of half-sandwich (η5-Cp*)Rh(III) cation with histidine containing peptides and their ternary species with (N,N) bidentate ligands. J. Inorg. Biochem..

[93] Enyedy É.A., Mészáros J.P., Dömötör O., Hackl C.M., Roller A., Keppler B.K., Kandioller W. (2015). Comparative solution equilibrium studies on pentamethylcyclopentadienyl rhodium complexes of 2,2′-bipyridine and ethylenediamine and their interaction with human serum albumin. J. Inorg. Biochem..

[94] Beck M.T., Nagypál I. (1990). Chemistry of Complex Equilibria, Joint Edition Published by Akadémiai Kiadó.

[95] Schwarzenbach G., Anderegg G. (1957). Die Verwendung der Quecksilberelektrode zur Bestimmung der Stabilitätskonstanten von Metallkomplexen. Helv. Chim. Acta.

[96] Harris W.R., Raymond K.N., Weitl F.L. (1981). Ferric Ion Sequestering Agents. 6. The Spectrophotometric and Potentiometric Evaluation of Sulfonated Tricatecholate Ligands. J. Am. Chem. Soc..

[97] Griffith D.M., Bíró L., Platts J.A., Müller-Bunz H., Farkas E., Buglyó P. (2012). Synthesis and solution behaviour of stable mono-, di- and trinuclear Pd(II) complexes of 2,5-pyridinedihydroxamic acid: X-ray crystal structure of a novel Pd(II) hydroxamato complex. Inorg. Chim. Acta.

[98] Bíró L., Farkas E., Buglyó P. (2010). Complex formation between Ru(η^6^-*p*-cym)(H_2_O)_3_]^2+^ and (O,O) donor ligands with biological relevance in aqueous solution. Dalton Trans..

[99] Enyedy É.A., Sija É., Jakusch T., Hartinger C.G., Kandioller W., Keppler B.K., Kiss T. (2013). Solution equilibria of anticancer ruthenium(II)-(η^6^-*p*-cymene)-hydroxy(thio)pyr(id)one complexes: Impact of sulfur vs. oxygen donor systems on the speciation and bioactivity. J. Inorg. Biochem..

[100] Enyedy É.A., Dömötör O., Hackl C.M., Roller A., Novak M.S., Jakupec A., Keppler B.K., Kandioller W. (2015). Solution equilibria and antitumor activities of pentamethylcyclopentadienyl rhodium complexes of picolinic acid and deferiprone. J. Coord. Chem..

[101] Mészáros J.P., Pape V.F.S., Szakács G., Németi G., Dénes M., Holczbauer T., May N.V., Enyedy É.A. (2021). Half-sandwich organometallic Ru and Rh complexes of (N,N) donor compounds: Effect of ligand methylation on solution speciation and anticancer activity. Dalton Trans..

[102] Dömötör O., Pape V.F.S., May N.V., Szakács G., Enyedy É.A. (2017). Comparative solution equilibrium studies of antitumor ruthenium(η^6^-*p*-cymene) and rhodium(η^5^-C_5_Me_5_) complexes of 8-hydroxyquinolines. Dalton Trans..

[103] Hackl C.M., Legina M.S., Pichler V., Schmidlehner M., Roller A., Dömötör O., Enyedy É.A., Jakupec M.A., Kandioller W., Keppler B.K. (2016). Thiomaltol-Based Organometallic Complexes with 1-Methylimidazole as Leaving Group: Synthesis, Stability, and Biological Behavior. Chem. Eur. J..

[104] Buglyó P., Parajdi-Losonczi P.L., Bényei A.C., Lihi N., Bíró L., Farkas E. (2017). Versatility of coordination modes in complexes of monohydroxamic acids with half-sandwich type ruthenium, rhodium, osmium and iridium cations. Chem. Select.

[105] Bruijnincx P.C.A., Sadler P.J. (2006). Controlling platinum, ruthenium and osmium reactivity for anticancer drug design. Adv. Inorg. Chem..

[106] Habtemariam A., Melchart M., Fernandez R., Parsons S., Oswald I.D., Parkin A., Fabbiani F.P., Davidson J.E., Dawson A., Aird R.E. (2006). Structure-activity relationships for cytotoxic ruthenium(II) arene complexes containing N,N-, N,O-, and O,O-chelating ligands. J. Med. Chem..

